# Wee1 inhibitor MK1775 sensitizes KRAS mutated NSCLC cells to sorafenib

**DOI:** 10.1038/s41598-017-18900-y

**Published:** 2018-01-17

**Authors:** Elisa Caiola, Roberta Frapolli, Michele Tomanelli, Rossana Valerio, Alice Iezzi, Marina C. Garassino, Massimo Broggini, Mirko Marabese

**Affiliations:** 1 Laboratory of Molecular Pharmacology, Department of Oncology, IRCCS - Istituto di Ricerche Farmacologiche “Mario Negri”, Milan, Italy; 2 Laboratory of Cancer Pharmacology, Department of Oncology, IRCCS - Istituto di Ricerche Farmacologiche “Mario Negri”, Milan, Italy; 3 Thoracic Oncology Unit, Department of Medical Oncology, Fondazione IRCCS Istituto Nazionale dei Tumori, Milan, Italy

## Abstract

Non-Small-Cell Lung Cancer (NSCLC) is a poorly chemosensitive tumor and targeted therapies are only used for about 15% of patients where a specific driving and druggable lesion is observed (EGFR, ALK, ROS). *KRAS* is one of the most frequently mutated genes in NSCLC and patients harboring these mutations do not benefit from specific treatments. Sorafenib, a multi-target tyrosine kinase inhibitor, was proposed as a potentially active drug in KRAS-mutated NSCLC patients, but clinical trials results were not conclusive. Here we show that the NSCLC cells’ response to sorafenib depends on the type of *KRAS* mutation. KRAS G12V cells respond less to sorafenib than the wild-type counterpart, *in vitro* and *in vivo*. To overcome this resistance, we used high-throughput screening with a siRNA library directed against 719 human kinases, and Wee1 was selected as a sorafenib response modulator. Inhibition of Wee1 by its specific inhibitor MK1775 in combination with sorafenib restored the *KRAS* mutated cells’ response to the multi-target tyrosine kinase inhibitor. This combination of the Wee1 inhibitor with sorafenib, if confirmed in models with different genetic backgrounds, might be worth investigating further as a new strategy for KRAS mutated NSCLC.

## Introduction

RAS are small GTPases proteins that play as molecular switches by coupling cell membrane growth factor receptors to intracellular signalling pathways^1^. *KRAS* mutations are the most frequent mutations (about 25%) in patients with non-small-cell lung cancer (NSCLC) and confer a poor prognosis for advanced disease^2,3^. *KRAS* mutations are point mutations resulting in the loss of intrinsic GTPase activity and consequently the deregulation of cell signals^4^. The RAS/MAPK pathway, together with the PI3K/AKT/mTOR cascade, is the major signalling network in cell proliferation and survival^5^. In the last ten years, a huge amount of work has focused on these pathways, and has resulted in a better understanding of the network. Unlike ALK and *EGFR* alterations, which can be targeted with specific drugs, so far there is no specific therapy for patients with *KRAS*-mutated tumors^6^.

Sorafenib is a multi-target tyrosine kinase inhibitor with anti-proliferative and anti-angiogenic activity^7^. It was primarily developed as an inhibitor of Raf proteins, but subsequent studies demonstrated that it also inhibits several other tyrosine kinase proteins. Sorafenib was reported to inhibit platelet-derived growth factor receptor-beta (PDGFRβ), vascular endothelial growth factor receptors 1, 2 and 3 (VEGFRs 1,2,3), c-KIT, RET, bRaf and Flt3^7^. In NSCLC patients sorafenib, alone or in addition to chemotherapy, has shown some activity^8,9^ although a randomized phase III trial in unselected NSCLC patients failed to show any benefit when it was added to cispaltin/gemcitabine in first line^10^.

The BATTLE study was a biomarker-based adaptively randomized trial in NSCLC patients refractory to previous therapy in which patients harboring a *KRAS*-mutated NSCLC received sorafenib. Although the results were not statistically significant, they suggested that patients with a *KRAS* mutation may benefit from sorafenib^11^.

In the same period, our laboratory reported that different *KRAS* mutations, according to the replaced bases, have different roles in drug responses, including sorafenib. Cells expressing G12V and G12C *KRAS* mutations were resistant to sorafenib^12^.

Further subgroup analyses of the BATTLE trial indicated that only specific *KRAS* mutations are associated with different drug responses. Patients harboring G12C and G12V *KRAS* mutations had significant lower progression-free survival than patients with all other KRAS mutants or the wild-type form^13^, confirming our previously findings on our isogenic system *in vitro*^12^.

Using our well-established system expressing different KRAS mutations, we investigated ways to overcome the resistance to sorafenib.

## Results

### *In vitro* response to sorafenib

**Figure 1:**
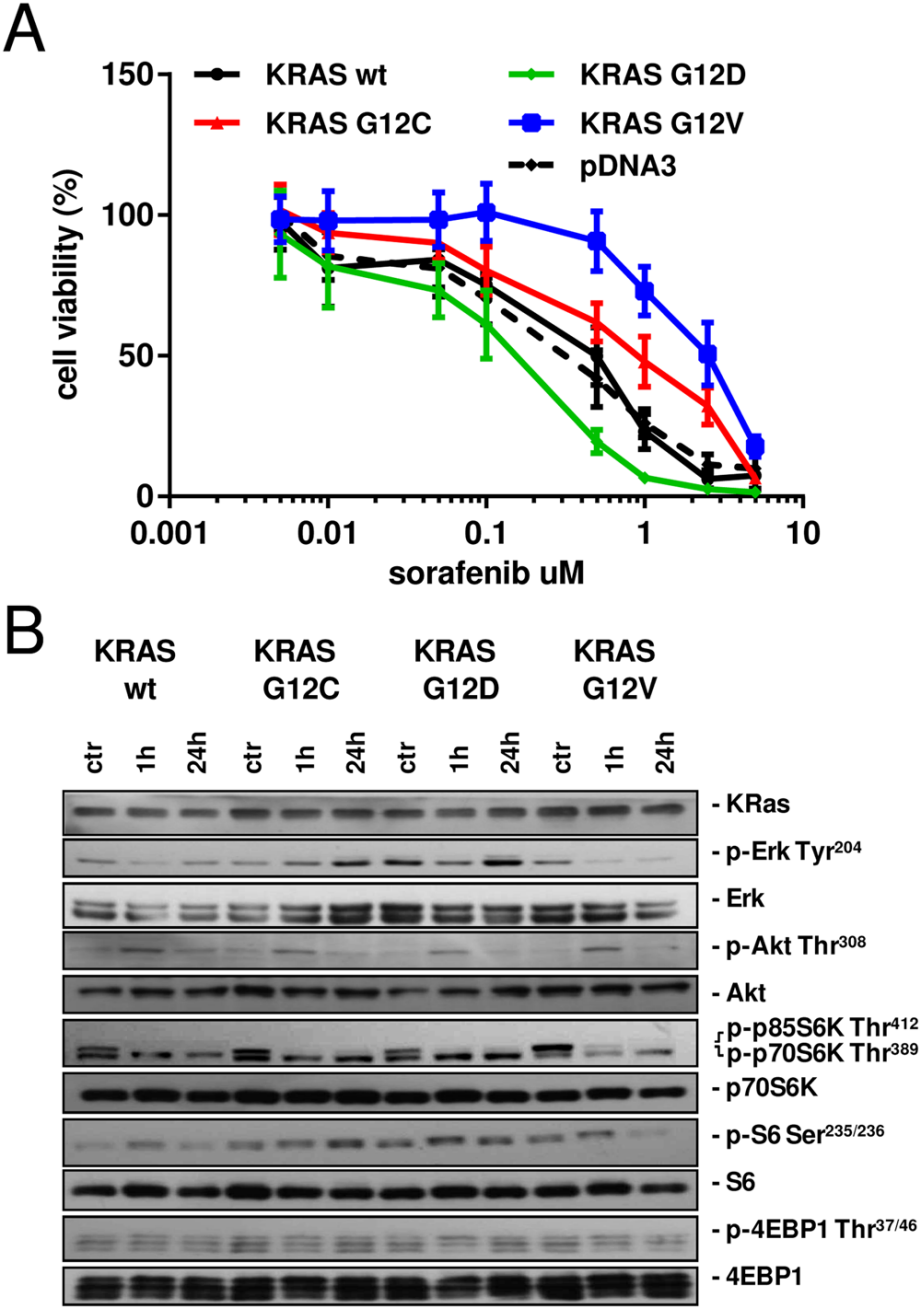
(**A**) Response of cells to sorafenib detected by MTS assay. The average of three independent experiments and SD are shown. (**B**) Representative Western blot analysis reporting the expression and the phosphorylation of different proteins belonging to the MAPK and PI3K pathways in the KRAS-expressing clones treated with sorafenib 1 uM, at the indicated time points.

Using isogenic NCI-H1299 derived clones expressing wild-type (wt), G12C, G12D or G12V variants of KRAS protein at comparable levels^12,14^, we determined the activity of sorafenib *in vitro*. The response of wt cells was comparable to the one of the clone transfected with the empty vector. The expression of the mutant form G12V induced less response than the KRAS wt protein. Other mutants, G12C and G12D, did not significantly change the sensitivity of the cells to the drug (Fig. 1A) with only a slight decrease (G12C) or slight increase (G12D) in response compared to wt KRAS expressing cells. IC50 from the mean curves indicated an approximately five-fold difference between the wt (IC50 = 0.5 uM) and the G12V KRAS-expressing clones (IC50 = 2.5 uM), while the G12C (IC50 = 0.8 uM) and the G12D (IC50 = 0.3 uM) clones had comparable IC50 to the wt cells.

### Evaluation of MAPK and PI3K signaling

To clarify the effects on pathways in the clones expressing different forms of KRAS, we examined the phosphorylation status of proteins belonging to PI3K and MAPK cascades at different times (1 and 24 h) after sorafenib 1 uM (Fig. 1B).

In the wt KRAS expressing clone, sorafenib induced a transient decrease of p-Erk 1 h after treatment start, and the starting phosphorylation level was restored after 24 h. The phosphorylated form of Erk accumulated in G12C KRAS cells during the 24 h of the experiment. In the G12D KRAS clone, Erk was less active 1 h after treatment and had returned to the untreated condition 24 h later. in G12V cells sorafenib drastically reduced the phosphorylation of Erk starting 1 h after treatment and this reduction persisted at least till 24 h. Sorafenib induced a transient increase of p-Akt(thr308) peaking around 1 h post-treatment in all the clones. By 24 h later, the levels of p-Akt were back to the untreated condition. The patterns of p-p70S6K(thr389) and p-p85S6K(thr412) were similar in all clones. p85S6K was inactivated 1 h after treatment and this persisted until 24 h while no modulation was detected for p70S6K. In the wt KRAS clone, p-S6(ser235/236) was activated 1 h after treatment and had returned to the untreated condition after 24 h. In the G12V KRAS clone no change was detected after 1 h for the same protein and it was de-phosphorylated only 24 h after treatment. In G12C and G12D-expressing cells, the levels of p-S6 were unchanged at all time points after sorafenib treatment. Sorafenib induced no major effect on p-4EBP1(thr37/46).

### *In vivo* sorafenib response and pharmacodynamics

**Figure 2:**
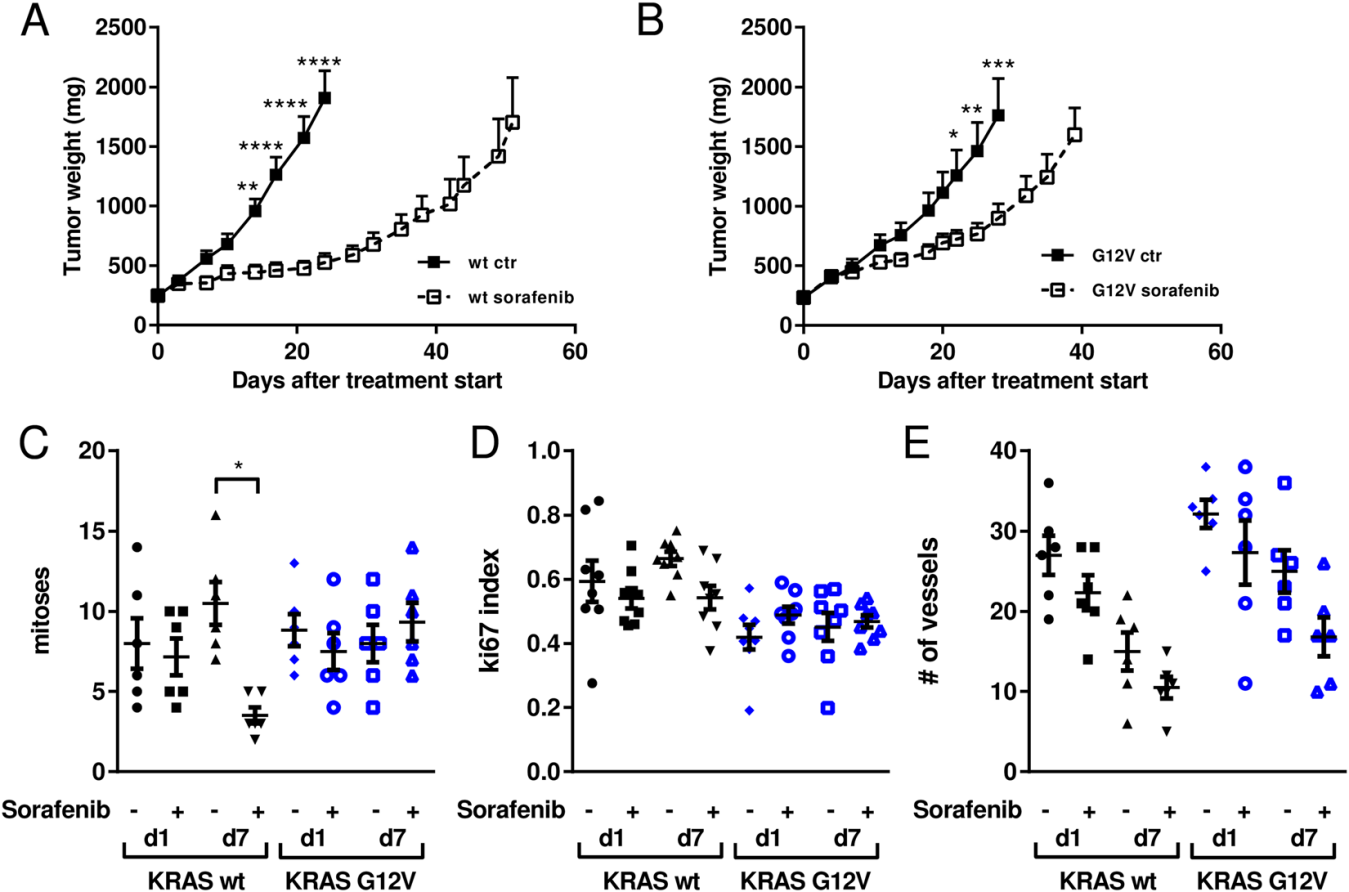
(**A**) Antitumor effects of 100 mg/kg of sorafenib daily in NCI-H1299 KRAS wt murine xenografts. *p < 0.05, **p < 0.01, ***p < 0.001. (**B**) Antitumor effects of 100 mg/kg of sorafenib daily in NCI-H1299 KRAS G12V murine xenografts. ****p < 0.0001 (**C**) Number of mitoses in six samples per group on days 1 and 7 after the last sorafenib dose in wt and G12V murine xenografts. *p < 0.05. (**D**) Ki-67 index in six samples per group on days 1 and 7 after the last sorafenib dose in wt and G12V murine xenografts. (**E**) Number of vessels in six samples per group on days 1 and 7 after the last sorafenib dose in wt and G12V murine xenografts.

To determine whether the sorafenib resistance of KRAS G12V cells *in vitro* was maintained *in vivo*, wt KRAS and KRAS G12V clones were xenotransplanted in nude mice. When the tumors reached approximately 150 mm^3^, mice were randomized and treated with sorafenib 100 mg/kg for 20 consecutive days. Following this treatment, the KRAS wt clone showed good sensitivity, with a best treated-over-control ratio (T/C) of 27% and an absolute growth delay (AGD) of 25 days (Fig. 2A). The tumors expressing KRAS G12V had a best T/C of only 51% and an AGD of 12 days (Fig. 2B). In the KRAS wt tumor-bearing mice two out of ten mice gave a complete response. These mice were monitored until day 100 and no relapse was observed.

Additional animals were included in each experimental group and used for pharmacodynamic investigations. After the end of the treatment period, animals were sacrificed 24 h (day 1) and 168 h (day 7) after their last sorafenib dose to determine changes in mitoses and vessels number. On day 7, KRAS wt tumors treated with sorafenib had a significant lower number of mitoses (p < 0.05) than the control group. None of the other experimental groups had differences in the number of mitoses compared to the vehicle-treated animals (Fig. 2C). A slight but not significant decrease was detected in the Ki-67 staining on day 7 in wt KRAS tumors in treated compared to control animals (Fig. 2D). A not significant decrease was detected in the number of vessels on days 1 and 7 in both wt and KRAS G12V tumors in treated compared to the control animals (Fig. 2E).

### High-throughput siRNA screening

To identify genes in synthetic lethality with sorafenib, which could be targeted to enhance the drug’s efficacy in resistant tumors, we performed a high-throughput screening with a siRNA library directed against 719 human protein kinases (Supplementary Table 1). Using the KRAS G12V-expressing clone, which was resistant in the previous experiments, we investigated the activity of siRNAs targeting the 719 kinases with or without sorafenib.

**Figure 3:**
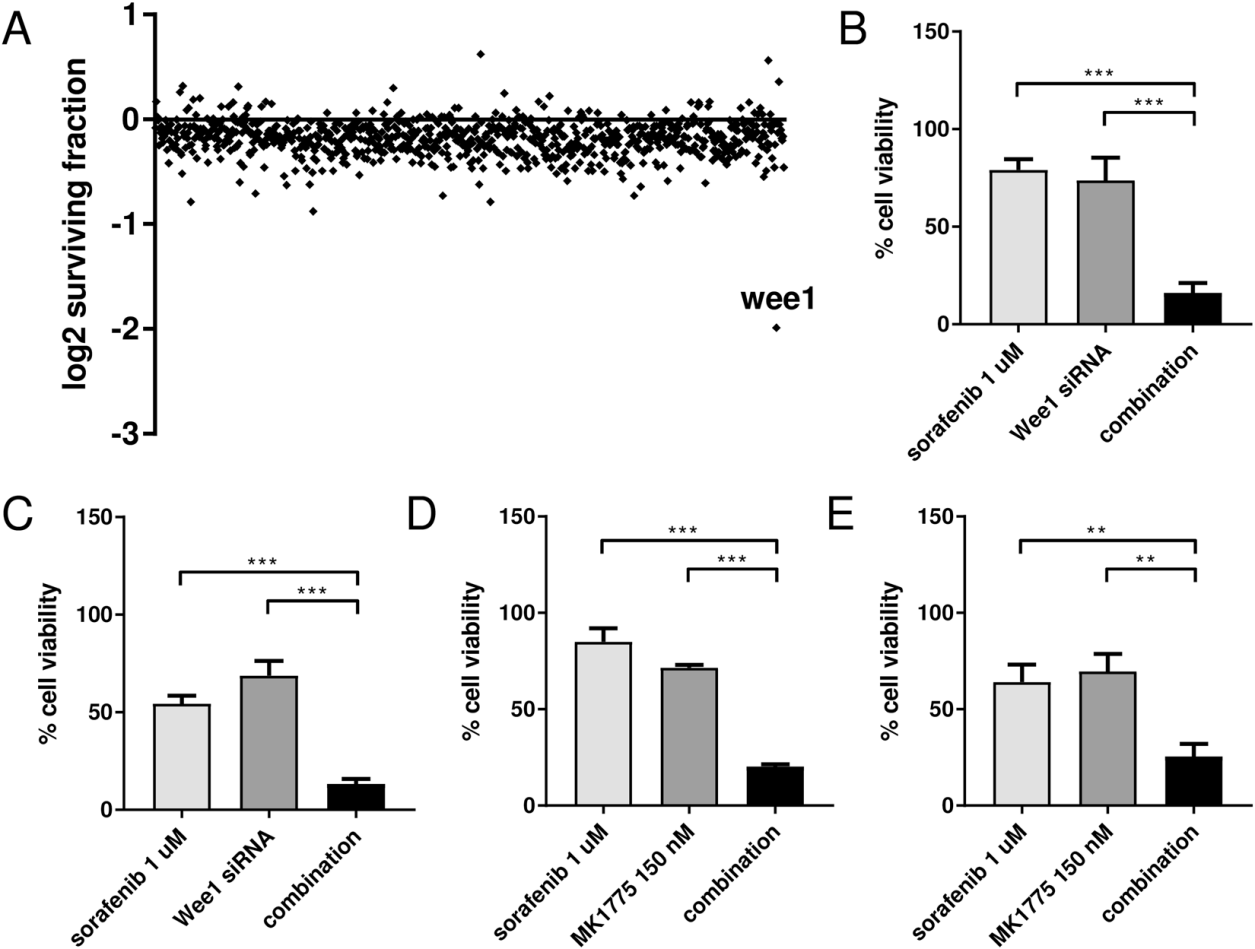
(**A**) Sensitivity of KRAS G12V cells treated with sorafenib 1 uM and transfected with each siRNA pool. The log2 of the surviving fraction is shown. (**B**) Response of G12V cells to sorafenib (1 uM), Wee1 esiRNA (30 nM) or the combination detected by MTS assay. The average of three independent experiments and SD are shown. ***p < 0.001. (**C**) Response of G12C cells to sorafenib (1 uM), Wee1 esiRNA (30 nM) or the combination detected by MTS assay. The average of three independent experiments and SD are shown. ***p < 0.001. (**D**) Response of G12V cells to sorafenib (1 uM), MK1775 (150 nM) or the combination detected by MTS assay. The average of three independent experiments and SD are shown. ***p < 0.001. (**E**) Response of G12C cells to sorafenib (1 uM), MK1775 (150 nM) or the combination detected by MTS assay. The average of three independent experiments and SD are shown. **p < 0.01.

The high-throughput screening identified Wee1 as a kinase potentially in synthetic lethality with sorafenib (Fig. 3A).

To validate Wee1 as possible target, we applied a different siRNA against Wee1 in combination with sorafenib in the G12V expressing clone. The Wee1 inhibition was able to sensitize G12V cells to sorafenib (Fig. 3B). The same approach was applied to the G12C clone to verify if the sensitization was specific for a peculiar type of KRAS mutation. Although the G12C clone was more responsive to the sorafenib treatment alone compared to the G12V cells, the combination with Wee1 siRNA enhanced the drug activity also in this setting (Fig. 3C).

Finally, we used a small molecule able to inhibit Wee1 in combination with sorafenib on KRAS mutant cells. In G12V cells, the treatments with sorafenib 1 uM and Wee1 inhibitor (MK1775) 150 nM as a single agents inhibited cell viability by 15% and 29%, respectively. The combination of the two drugs at the concentrations used as single agents had a strongly cytotoxic effect, reaching 80% inhibition of cell viability (Fig. 3D). The same result was obtained by treating the G12C clone indicating that this approach could be applied to cells harboring different KRAS mutations (Fig. 3E).

## Discussion

Sorafenib is a multi-kinase inhibitor with numerous properties such as anti-angiogenic, pro-apoptotic and anti-proliferative effects. Preclinical studies show that this molecule acts through various molecular targets including bRaf (both wt and mutated), PDGFRβ, cKit, Flt3 and the VEGFR family^7^.

The MAPK pathway is upregulated in KRAS-mutated NSCLC^15^ and in different types of tumor sorafenib can switch off MAPK signaling through Raf inhibition, as evidenced by reduced pErk levels^16^. For this reason, sorafenib was presumed to be active in tumors harboring *KRAS* mutations.

The BATTLE trial was a biomarker-based adaptively randomized study that treated 158 pretreated NSCLC patients with erlotinib, vandetanib, erlotinib + bexarotene, or sorafenib according to predefined biomarkers including the *KRAS* mutational status. Although the trial result was not significant, patients with a mutated form of KRAS seemed to benefit from sorafenib treatment^11^. However, in a following small single-center study specifically aimed at evaluating the response to sorafenib, the authors did not find any benefit in patients with *KRAS* mutation^17^. Later, a subgroup analysis in the MISSION trial did not detect any benefit for KRAS-mutated patients treated with sorafenib^18^.

The lack of positive results in these studies might be due to having considered the *KRAS* general status, while the different amino acid substitutions induced by a pool of *KRAS* mutations in patients may have different impacts on the outcome^11,12^. Our group has shed light on the possibility that the expression of a specific KRAS mutated protein may induce different patterns of sensitivity to different drugs, including sorafenib. For example, NSCLC cells expressing the KRAS G12D mutation responded well to sorafenib while the G12V mutation was associated with resistance, suggesting that the different *KRAS* mutations interact differently with the treatment^12^.

These data were confirmed one year later by Ihle and co-workers who analyzed the BATTLE trial data. They showed that patients with G12C and G12V KRAS NSCLCs had a shorter progression-free survival than patients with other types of *KRAS* mutations treated with sorafenib^13^.

We have now confirmed *in vivo*, using xenograft models, that the G12V KRAS mutation gives a weak response to sorafenib compared to models expressing the wt KRAS. This lower drug activity seems not to be related to the antiangiogenic activity of sorafenib, as the numbers of vessels after treatment were similarly reduced in KRAS wt and G12V KRAS mutated cells. In good correlation with what the *in vitro* findings, there seems to be a direct effect on cell growing ability.

In addition, the novelty of the present work is that a synthetic lethality approach was applied to our NSCLC system as a way to enhance sorafenib activity. Our high-throughput siRNA screening targeting the mammalian kinome pointed to Wee1 as an enzyme to target in order to potentiate sorafenib’s activity in cells harboring the G12V KRAS mutation. Previously published data supported the idea that KRAS mutant cells may be more sensitive to the inhibition of G2/M regulators. Luo and co-workers highlighted the possibility that Ras mutants cells are characterized by mitotic stress and the interference of polo-like kinase 1 could exacerbate the mitotic stress resulting in cell death^19^. The importance of the mitosis regulation in Ras mutant cells was confirmed by using paclitaxel alone^19^ or in combination with sorafenib^20^.

Wee1 is a kinase that acts as a mitotic inhibitor in the intricate network regulating the G2 phase progression in the cell cycle. Wee1 and the phosphatase CDC25 are the main controllers for the mitosis process^21^.

Wee1, like many other kinases, has been described as a potential target for cancer therapy, given its deregulation in tumors. Studies describing human cancers with increased Wee1 expression have been reported^22–25^. However, several other publications have reported a lack of Wee1 expression in human cancers^26–28^.

Cancer cells deficient for p53 signaling show genomic instability and in general need Wee1 for survival during mitosis. In this condition, Wee1 plays the role of a cancer-conserving oncogene and inhibition of its activity may be exploited to sensitize cells toward combinations with DNA-damaging therapy^21^.

The isogenic system we used here harbors an impaired p53 and the activity of Wee1 may be essential for safe mitosis. In addition, we recently described a link between KRAS and the DNA repair machinery^29^. Cells harboring a specific *KRAS* mutation have dysregulation of the Base Excision Repair (BER) pathway. High BER activity due to DNA polymerase beta (polβ) up-regulation was associated with mutated KRAS and cisplatin resistance^14^. Furthermore, studies aimed at uncovering unique vulnerabilities of RAS-driven tumors have identified a number of genes that mediate mutant RAS cancer dependence on stress by mitigating mechanisms including DNA damage^29^.

In the light of these considerations, the absence of a proficient p53 and the particular vulnerability of KRAS mutated tumors to dysregulated DNA damage repair mechanisms, do suggest that the combination of the Wee1 inhibitor with sorafenib might be useful new strategy for this sub-population of cancers but also for RET rearranged cells as reported by Levinson *et al*.^30^.

## Methods

### Cell cultures, siRNA and drugs

NCI-H1299 derived clones were grown in RPMI-1640 medium including 500 µg/mL of G418 (Gibco). Clones were obtained by transfecting the NCI-H1299 cell line with the expression plasmids encoding for the mutant G12C, G12D and G12V KRAS and the wt KRAS, used as control. Details of transfection, KRAS protein expression and activation are reported in our previous paper^14^. Cells are routinely tested by PCR for mycoplasma contamination and authenticated with the PowerPlex 16 HS System (Promega) every six months by comparing the STR profiles with those deposited in ATCC and/or DSMZ databases. esiRNA were purchased by Sigma Aldrich and transfected by Lipofectamine 2000 (Invitrogen). Sorafenib and MK1775 (Selleckchem) DMSO stock solutions were dissolved in medium just before use. The MTS assays (Promega) were done as described in^14^. Survival curves were plotted as percentages of untreated controls, with at least six replicates for each time point. The mean and SD of at least three independent experiments are presented.

### Western blotting analyses

Proteins were extracted and visualized as reported in^14^. Immunoblotting was carried out with the following antibodies: anti-p70S6K(Thr389) #9206, anti-p70S6K #9202, anti-S6(Ser235/236) ribosomal protein #2211, anti-S6 ribosomal protein 2217#, anti-4E-BP1(Thr37/46) #2855, anti-4E-BP1 #9644 provided by Cell Signalling Technology. Anti-Erk #sc94, anti-Erk(Tyr204) #sc7383, anti-KRAS #sc30 were obtained from Santa Cruz Biotechnology.

### Immunohistochemistry analyses

Tumor samples were fixed in 10% formalin neutral buffer, routinely processed for histological examination and embedded in paraffin. Hematoxylin and Eosin (H&E) staining was done for morphological examination. Immunoperoxidase staining was done using an ordinary biotin-streptavidin method. Antigen retrieval was obtained by pressure-cooking in 0.01 M Citrate buffer (pH 6) for 3 minutes. We used a Vectastain ABC kit (Vector, Burlingame, CA) and AEC (Carbazol) as chromogen. The sections were then lightly counterstained with hematoxylin. For each immunohistochemistry staining we did an additional stain without primary antibody in parallel as negative control.

Microvessel density (MD) through CD31 immunohistochemistry was recorded on 4 μm sections from each tumor xenograft, which were immunostained with a primary rat monoclonal antibody against CD31 (PECAM) antigen (Dianova; clone SZ31). The number of CD31-positive vascular outlines were counted using the ImageJ analysis program (http://rsb.info.nih.gov/ij/) in three 200× microscopic fields randomly selected throughout the neoplastic tissue.

Evaluation of proliferative activity was assessed through Ki-67 staining. To assess the extent of the proliferative activity, 4 μm sections from each tumor xenograft were immunostained with a primary rabbit monoclonal antibody against Ki-67 antigen (LabVision; #RM-9106-S). For each sample, serial sections incubated with an irrelevant primary antibody produced in rabbit served as negative controls. The number of Ki67-positive and Ki-67-negative tumor cell nuclei were counted using the ImageJ analysis program in 4 400× microscopic fields randomly selected throughout the neoplastic tissue. Ki-67 index was calculated as Ki-67 positive cells/Ki-67 negative cells.

### *In vivo* experiments

Six-week-old female nude Foxn1 mice (≈25 g, Harlan Laboratories, Italy) were housed at constant temperature and humidity, according to institutional guidelines. Protocols were approved by the Ethics Committee of the IRCCS-Istituto di Ricerche Farmacologiche Mario Negri (Italy), in compliance with national (D.lgs 26/2014; Authorisation no. 19/2008-A issued March 6, 2008 by Ministry of Health) and international laws (EU Directive 2010/63/EU). Mice were injected s.c. with 200 ul of cell suspension containing 10^7^ cells. When the average tumor weights reached about 150 mg (excluding animals with tumors <100 mg or >400 mg in weight), mice were stratified and distributed in experimental groups in order to obtain similar means and SEM among groups. Each group comprised seven mice. Groups were allocated to treatments in blind. Sorafenib was given orally by gavage at the dose of 100 mg/kg for 20 days consecutively. The investigator who did the *in vivo* studies was not informed about the *in vitro* results regarding sorafenib citotoxicity. Treatment efficacy was evaluated using the optimal T/C% and absolute growth delay (AGD). T/C% was calculated as 100 × T/C where T and C were the mean tumor volumes in the treated and control groups, respectively. A T/C <42% is considered the minimum for activity^31^. AGD was calculated as the difference between the average times required to reach a tumor volume of 1 g in treated and control groups. Tumor weight was compared at each time point using two-way ANOVA followed by Bonferroni’s a posteriori test on log-transformed data.

### High-throughput siRNA screening

The Mission siRNA Human Kinase Panel (Sigma Aldrich) was employed. This library includes three different siRNAs for each of the 719 different targets. The three siRNAs targeting the same gene were pooled at equal molarity for screening. Procedures were performed by using the automated liquid handling system Janus (PerkinElmer). Briefly, on day 1 cells were seeded in 384-well plates. On day 2, cells were transfected y using Lipofectamine 2000 (Invitrogen) with the siRNA pool of each target or a scramble siRNA. On day 3 cells were treated with sorafenib 1 uM. Seventy-two hours after treatment started cell survival was analyzed by the MTS assay (Promega). The sensitivity to sorafenib for each siRNA pool transfected was assessed as the fraction affected using the following formula: log2(mean replica of samples treated with sorafenib and library siRNAs) – log2(mean replica of samples treated with vehicle and library siRNA). Two independent experiments were run.

### Statistical analyses

Statistical analyses were done using GraphpadPrism version 6.05. Differences between groups were considered significant when the p-values were ≤0.05.

## Electronic supplementary material

Supplementary Table 1
