# Supplementary material for: Wee1 inhibitor MK1775 sensitizes KRAS mutated NSCLC cells to sorafenib
Source: Sci Rep. 2018 Jan 17;8:948. doi: 10.1038/s41598-017-18900-y (PMC5772438; doi:10.1038/s41598-017-18900-y)
Supplement: Supplementary file 1 — Supplementary Table 1 [file 41598_2017_18900_MOESM1_ESM.pdf]

Wee1 inhibitor MK1775 sensitizes KRAS mutated NSCLC cells to sorafenib

Elisa Caiola[1], Roberta Frapolli[2], Michele Tomanelli[1], Rossana Valerio[1], Alice Iezzi[1], Marina C. Garassino[3], Massimo Broggini[1] and Mirko Marabese[1]*

[1]Laboratory of Molecular Pharmacology, Department of Oncology, IRCCS - Istituto di Ricerche Farmacologiche "Mario Negri", Milan, Italy.

[2]Laboratory of Cancer Pharmacology, Department of Oncology, IRCCS - Istituto di Ricerche Farmacologiche "Mario Negri", Milan, Italy.

[3]Thoracic Oncology Unit, Department of Medical Oncology, Fondazione IRCCS Istituto Nazionale dei Tumori, Milan, Italy.

MB and MM are co-last authors

*Corresponding author

Laboratory of Molecular Pharmacology, Department of Oncology, IRCCS - Istituto di Ricerche Farmacologiche Mario Negri, via La Masa 19, 20156 Milan, Italy. Phone number +39 0239014236 Fax number +39 0239014734 E-mail mirko.marabese@marionegri.it

Symbol	Accession #	Gene ID	Gene Description
AAK1	NM_014911	22848	AP2 associated kinase 1
AATK	NM_001080395	9625	apoptosis-associated tyrosine kinase
AATK	XM_001714082	9625	apoptosis-associated tyrosine kinase
AATK	XM_927215	9625	apoptosis-associated tyrosine kinase
ABCC1	NM_004996	4363	ATP-binding cassette, sub-family C (CFTR/MRP), member 1
ABL1	NM_005157	25	c-abl oncogene 1, receptor tyrosine kinase
ABL2	NM_001100108	27	v-abl Abelson murine leukemia viral oncogene homolog 2 (arg, Abelson-relate
ABL2	NM_005158	27	v-abl Abelson murine leukemia viral oncogene homolog 2 (arg, Abelson-relate
ABL2	NM_007314	27	v-abl Abelson murine leukemia viral oncogene homolog 2 (arg, Abelson-relate
ACVR1	NM_001105	90	activin A receptor, type I
ACVR1B	NM_004302	91	activin A receptor, type IB
ACVR1C	NM_001111031	130399	activin A receptor, type IC
ACVR1C	NM_001111032	130399	activin A receptor, type IC
ACVR1C	NM_145259	130399	activin A receptor, type IC
ACVR2A	NM_001616	92	activin A receptor, type IIA
ACVR2B	NM_001106	93	activin A receptor, type IIB
ACVRL1	NM_000020	94	activin A receptor type II-like 1
ADCK1	NM_020421	57143	aarF domain containing kinase 1
ADCK2	NM_052853	90956	aarF domain containing kinase 2
ADCK4	NM_024876	79934	aarF domain containing kinase 4
ADCK5	NM_174922	203054	aarF domain containing kinase 5
ADK	NM_001123	132	adenosine kinase
ADRBK1	NM_001619	156	adrenergic, beta, receptor kinase 1
ADRBK2	NM_005160	157	adrenergic, beta, receptor kinase 2
AGK	NM_018238	55750	acylglycerol kinase
AK1	NM_000476	203	adenylate kinase 1
AK2	NM_001625	204	adenylate kinase 2
AK3	NM_016282	50808	adenylate kinase 3
AK3L1	NM_001005353	205	adenylate kinase 3-like 1
AK5	NM_012093	26289	adenylate kinase 5
AK7	NM_152327	122481	adenylate kinase 7
AKAP13	NM_006738	11214	A kinase (PRKA) anchor protein 13
AKT1	NM_001014431	207	v-akt murine thymoma viral oncogene homolog 1
AKT2	NM_001626	208	v-akt murine thymoma viral oncogene homolog 2
AKT3	NM_005465	10000	v-akt murine thymoma viral oncogene homolog 3 (protein kinase B, gamma)
ALDH18A1	NM_001017423	5832	aldehyde dehydrogenase 18 family, member A1
ALDH18A1	NM_002860	5832	aldehyde dehydrogenase 18 family, member A1
ALK	NM_004304	238	anaplastic lymphoma receptor tyrosine kinase
ALPK1	NM_001102406	80216	alpha-kinase 1
ALPK1	NM_025144	80216	alpha-kinase 1
ALPK2	NM_052947	115701	alpha-kinase 2
ALPK3	NM_020778	57538	alpha-kinase 3
AMHR2	NM_020547	269	anti-Mullerian hormone receptor, type II
ANKK1	NM_178510	255239	ankyrin repeat and kinase domain containing 1
ARAF	NM_001654	369	v-raf murine sarcoma 3611 viral oncogene homolog
ATM	NM_000051	472	ataxia telangiectasia mutated

ATM	NM_138292	472	ataxia telangiectasia mutated
ATR	NM_001184	545	ataxia telangiectasia and Rad3 related
AURKA	NM_003600	6790	aurora kinase A
AURKB	NM_004217	9212	aurora kinase B
AURKC	NM_001015878	6795	aurora kinase C
AXL	NM_001699	558	AXL receptor tyrosine kinase
BAIAP2	NM_006340	10458	BAI1-associated protein 2
BCKDK	NM_001122957	10295	branched chain ketoacid dehydrogenase kinase
BCKDK	NM_005881	10295	branched chain ketoacid dehydrogenase kinase
BCR	NM_004327	613	breakpoint cluster region
BLK	NM_001715	640	B lymphoid tyrosine kinase
BMP2K	NM_017593	55589	BMP2 inducible kinase
BMPR1A	NM_004329	657	bone morphogenetic protein receptor, type IA
BMPR1B	NM_001203	658	bone morphogenetic protein receptor, type IB
BMPR2	NM_001204	659	bone morphogenetic protein receptor, type II (serine/threonine kinase)
BMX	NM_001721	660	BMX non-receptor tyrosine kinase
BRAF	NM_004333	673	v-raf murine sarcoma viral oncogene homolog B1
BRD2	NM_001113182	6046	bromodomain containing 2
BRD2	NM_005104	6046	bromodomain containing 2
BRD3	NM_007371	8019	bromodomain containing 3
BRD4	NM_014299	23476	bromodomain containing 4
BRDT	NM_001726	676	bromodomain, testis-specific
BRSK1	NM_032430	84446	BR serine/threonine kinase 1
BRSK2	NM_003957	9024	BR serine/threonine kinase 2
BTK	NM_000061	695	Bruton agammaglobulinemia tyrosine kinase
BUB1	NM_004336	699	budding uninhibited by benzimidazoles 1 homolog (yeast)
BUB1B	NM_001211	701	budding uninhibited by benzimidazoles 1 homolog beta (yeast)
C1orf57	NM_032324	84284	chromosome 1 open reading frame 57
C6orf199	NM_145025	221264	adenylate kinase domain containing 1
C9orf96	NM_153710	169436	chromosome 9 open reading frame 96
CABC1	NM_020247	56997	chaperone, ABC1 activity of bc1 complex homolog (S. pombe)
CALM1	NM_006888	801	calmodulin 1 (phosphorylase kinase, delta)
CALM2	NM_001743	805	calmodulin 2 (phosphorylase kinase, delta)
CALM3	NM_005184	808	calmodulin 3 (phosphorylase kinase, delta)
CAMK1	NM_003656	8536	calcium/calmodulin-dependent protein kinase I
CAMK1D	NM_020397	57118	calcium/calmodulin-dependent protein kinase ID
CAMK1G	NM_020439	57172	calcium/calmodulin-dependent protein kinase IG
CAMK2A	NM_015981	815	calcium/calmodulin-dependent protein kinase II alpha
CAMK2B	NM_001220	816	calcium/calmodulin-dependent protein kinase II beta
CAMK2B	NM_172078	816	calcium/calmodulin-dependent protein kinase II beta
CAMK2B	NM_172079	816	calcium/calmodulin-dependent protein kinase II beta
CAMK2B	NM_172082	816	calcium/calmodulin-dependent protein kinase II beta
CAMK2D	NM_001221	817	calcium/calmodulin-dependent protein kinase II delta
CAMK2G	NM_001222	818	calcium/calmodulin-dependent protein kinase II gamma
CAMK2G	NM_172172	818	calcium/calmodulin-dependent protein kinase II gamma
CAMK4	NM_001744	814	calcium/calmodulin-dependent protein kinase IV
CAMKK1	NM_032294	84254	calcium/calmodulin-dependent protein kinase kinase 1, alpha

CAMKK2	NM_006549	10645	calcium/calmodulin-dependent protein kinase kinase 2, beta
CAMKV	NM_024046	79012	CaM kinase-like vesicle-associated
CARD14	NM_024110	79092	caspase recruitment domain family, member 14
CASK	NM_001126054	8573	calcium/calmodulin-dependent serine protein kinase (MAGUK family)
CASK	NM_003688	8573	calcium/calmodulin-dependent serine protein kinase (MAGUK family)
CCRK	NM_001039803	23552	cell cycle related kinase
CCT2	NM_006431	10576	chaperonin containing TCP1, subunit 2 (beta)
CDC14B	NM_001077181	8555	CDC14 cell division cycle 14 homolog B (S. cerevisiae)
CDC14B	NM_033331	8555	CDC14 cell division cycle 14 homolog B (S. cerevisiae)
CDC2	NM_001786	983	cell division cycle 2, G1 to S and G2 to M
CDC2L1	NM_001787	984	cell division cycle 2-like 1 (PITSLRE proteins)
CDC2L1	NM_033489	984	cell division cycle 2-like 1 (PITSLRE proteins)
CDC2L2	NM_033527	985	cell division cycle 2-like 2 (PITSLRE proteins)
CDC2L2	NM_033528	985	
CDC2L2	NM_033532	985	
CDC2L2	NM_033534	985	
CDC2L2	NM_033536	985	
CDC2L2	NM_033537	985	
CDC2L5	NM_003718	8621	cell division cycle 2-like 5 (cholinesterase-related cell division controller)
CDC2L6	NM_015076	23097	cell division cycle 2-like 6 (CDK8-like)
CDC42BPA	NM_003607	8476	CDC42 binding protein kinase alpha (DMPK-like)
CDC42BPB	NM_006035	9578	CDC42 binding protein kinase beta (DMPK-like)
CDC42BPG	NM_017525	55561	CDC42 binding protein kinase gamma (DMPK-like)
CDC7	NM_003503	8317	cell division cycle 7 homolog (S. cerevisiae)
CDK10	NM_001098533	8558	cyclin-dependent kinase 10
CDK10	NM_003674	8558	cyclin-dependent kinase 10
CDK10	NM_052988	8558	cyclin-dependent kinase 10
CDK2	NM_001798	1017	cyclin-dependent kinase 2
CDK3	NM_001258	1018	cyclin-dependent kinase 3
CDK4	NM_000075	1019	cyclin-dependent kinase 4
CDK5	NM_004935	1020	cyclin-dependent kinase 5
CDK5R1	NM_003885	8851	cyclin-dependent kinase 5, regulatory subunit 1 (p35)
CDK5R2	NM_003936	8941	cyclin-dependent kinase 5, regulatory subunit 2 (p39)
CDK6	NM_001259	1021	cyclin-dependent kinase 6
CDK7	NM_001799	1022	cyclin-dependent kinase 7
CDK8	NM_001260	1024	cyclin-dependent kinase 8
CDK9	NM_001261	1025	cyclin-dependent kinase 9
CDKL1	NM_004196	8814	cyclin-dependent kinase-like 1 (CDC2-related kinase)
CDKL2	NM_003948	8999	cyclin-dependent kinase-like 2 (CDC2-related kinase)
CDKL3	NM_001113575	51265	cyclin-dependent kinase-like 3
CDKL3	NM_016508	51265	cyclin-dependent kinase-like 3
CDKL4	NM_001009565	344387	cyclin-dependent kinase-like 4
CDKL5	NM_001037343	6792	cyclin-dependent kinase-like 5
CERK	NM_022766	64781	ceramide kinase
CERK	NM_182661	64781	ceramide kinase
CERKL	NM_001030311	375298	ceramide kinase-like
CERKL	NM_001030314	375298	ceramide kinase-like

CHEK1	NM_001114121	1111	CHK1 checkpoint homolog (S. pombe)
CHEK1	NM_001274	1111	CHK1 checkpoint homolog (S. pombe)
CHEK2	NM_001005735	11200	CHK2 checkpoint homolog (S. pombe)
CHKA	NM_001277	1119	choline kinase alpha
CHKB	NM_005198	1120	choline kinase beta
CHKB	NM_152253	1120	choline kinase beta
CHUK	NM_001278	1147	conserved helix-loop-helix ubiquitous kinase
CIT	NM_007174	11113	citron (rho-interacting, serine/threonine kinase 21)
CKB	NM_001823	1152	creatine kinase, brain
CKM	NM_001824	1158	creatine kinase, muscle
CKMT1B	NM_020990	1159	creatine kinase, mitochondrial 1B
CKMT2	NM_001099735	1160	creatine kinase, mitochondrial 2 (sarcomeric)
CKMT2	NM_001825	1160	creatine kinase, mitochondrial 2 (sarcomeric)
CKS1B	NM_001826	1163	CDC28 protein kinase regulatory subunit 1B
CKS2	NM_001827	1164	CDC28 protein kinase regulatory subunit 2
CLK1	NM_001024646	1195	CDC-like kinase 1
CLK1	NM_004071	1195	CDC-like kinase 1
CLK2	NM_001291	1196	CDC-like kinase 2
CLK2	NM_003993	1196	CDC-like kinase 2
CLK3	NM_001292	1198	CDC-like kinase 3
CLK3	NM_003992	1198	CDC-like kinase 3
CLK4	NM_020666	57396	CDC-like kinase 4
CMPK1	NM_016308	51727	cytidine monophosphate (UMP-CMP) kinase 1, cytosolic
COASY	NM_001042529	80347	Coenzyme A synthase
COASY	NM_001042531	80347	Coenzyme A synthase
COASY	NM_025233	80347	Coenzyme A synthase
COL4A3BP	NM_005713	10087	collagen, type IV, alpha 3 (Goodpasture antigen) binding protein
CPT1B	NM_004377	1375	carnitine palmitoyltransferase 1B (muscle)
CRKL	NM_005207	1399	v-crk sarcoma virus CT10 oncogene homolog (avian)-like
CRKRS	NM_015083	51755	Cdc2-related kinase, arginine/serine-rich
CRKRS	NM_016507	51755	Cdc2-related kinase, arginine/serine-rich
CSF1R	NM_005211	1436	colony stimulating factor 1 receptor
CSK	NM_001127190	1445	c-src tyrosine kinase
CSK	NM_004383	1445	c-src tyrosine kinase
CSNK1A1	NM_001025105	1452	casein kinase 1, alpha 1
CSNK1A1L	NM_145203	122011	casein kinase 1, alpha 1-like
CSNK1D	NM_001893	1453	casein kinase 1, delta
CSNK1E	NM_001894	1454	casein kinase 1, epsilon
CSNK1G1	NM_001011664	53944	casein kinase 1, gamma 1
CSNK1G1	NM_022048	53944	casein kinase 1, gamma 1
CSNK1G2	NM_001319	1455	casein kinase 1, gamma 2
CSNK1G3	NM_001031812	1456	casein kinase 1, gamma 3
CSNK2A1	NM_001895	1457	casein kinase 2, alpha 1 polypeptide
CSNK2A1	NM_177560	1457	casein kinase 2, alpha 1 polypeptide
CSNK2A2	NM_001896	1459	casein kinase 2, alpha prime polypeptide
CSNK2B	NM_001320	1460	casein kinase 2, beta polypeptide
DAK	NM_015533	26007	dihydroxyacetone kinase 2 homolog (S. cerevisiae)

DAPK1	NM_004938	1612	death-associated protein kinase 1
DAPK2	NM_014326	23604	death-associated protein kinase 2
DAPK3	NM_001348	1613	death-associated protein kinase 3
DCK	NM_000788	1633	deoxycytidine kinase
DCLK1	NM_004734	9201	doublecortin-like kinase 1
DCLK2	NM_001040260	166614	doublecortin-like kinase 2
DCLK3	NM_033403	85443	doublecortin-like kinase 3
DCLK3	XM_047355	85443	doublecortin-like kinase 3
DDR1	NM_001954	780	discoidin domain receptor tyrosine kinase 1
DDR2	NM_001014796	4921	discoidin domain receptor tyrosine kinase 2
DGKA	NM_001345	1606	diacylglycerol kinase, alpha 80kDa
DGKB	NM_004080	1607	diacylglycerol kinase, beta 90kDa
DGKD	NM_003648	8527	diacylglycerol kinase, delta 130kDa
DGKE	NM_003647	8526	diacylglycerol kinase, epsilon 64kDa
DGKG	NM_001080744	1608	diacylglycerol kinase, gamma 90kDa
DGKG	NM_001346	1608	diacylglycerol kinase, gamma 90kDa
DGKH	NM_152910	160851	diacylglycerol kinase, eta
DGKI	NM_004717	9162	diacylglycerol kinase, iota
DGKQ	NM_001347	1609	diacylglycerol kinase, theta 110kDa
DGKZ	NM_001105540	8525	diacylglycerol kinase, zeta 104kDa
DGKZ	NM_003646	8525	diacylglycerol kinase, zeta 104kDa
DGKZ	NM_201533	8525	diacylglycerol kinase, zeta 104kDa
DGUOK	NM_080916	1716	deoxyguanosine kinase
DLG1	NM_001098424	1739	discs, large homolog 1 (Drosophila)
DLG1	NM_004087	1739	discs, large homolog 1 (Drosophila)
DLG2	NM_001364	1740	discs, large homolog 2 (Drosophila)
DLG3	NM_020730	1741	discs, large homolog 3 (Drosophila)
DLG3	NM_021120	1741	discs, large homolog 3 (Drosophila)
DLG4	NM_001365	1742	discs, large homolog 4 (Drosophila)
DLG5	NM_004747	9231	discs, large homolog 5 (Drosophila)
DMPK	NM_001081560	1760	dystrophia myotonica-protein kinase
DMPK	NM_004409	1760	dystrophia myotonica-protein kinase
DTYMK	NM_012145	1841	deoxythymidylate kinase (thymidylate kinase)
DYRK1A	NM_001396	1859	dual-specificity tyrosine-(Y)-phosphorylation regulated kinase 1A
DYRK1A	NM_101395	1859	dual-specificity tyrosine-(Y)-phosphorylation regulated kinase 1A
DYRK1B	NM_004714	9149	dual-specificity tyrosine-(Y)-phosphorylation regulated kinase 1B
DYRK1B	NM_006483	9149	dual-specificity tyrosine-(Y)-phosphorylation regulated kinase 1B
DYRK2	NM_003583	8445	dual-specificity tyrosine-(Y)-phosphorylation regulated kinase 2
DYRK3	NM_001004023	8444	dual-specificity tyrosine-(Y)-phosphorylation regulated kinase 3
DYRK4	NM_003845	8798	dual-specificity tyrosine-(Y)-phosphorylation regulated kinase 4
EEF2K	NM_013302	29904	eukaryotic elongation factor-2 kinase
EGFR	NM_005228	1956	epidermal growth factor receptor (erythroblastic leukemia viral (v-erb-b) oncog
EIF2AK1	NM_014413	27102	eukaryotic translation initiation factor 2-alpha kinase 1
EIF2AK2	NM_002759	5610	eukaryotic translation initiation factor 2-alpha kinase 2
EIF2AK3	NM_004836	9451	eukaryotic translation initiation factor 2-alpha kinase 3
EIF2AK4	NM_001013703	440275	eukaryotic translation initiation factor 2 alpha kinase 4
EPHA1	NM_005232	2041	EPH receptor A1

EPHA10	NM_001004338	284656	EPH receptor A10
EPHA10	NM_001099439	284656	EPH receptor A10
EPHA10	NM_173641	284656	EPH receptor A10
EPHA2	NM_004431	1969	EPH receptor A2
EPHA3	NM_005233	2042	EPH receptor A3
EPHA4	NM_004438	2043	EPH receptor A4
EPHA5	NM_004439	2044	EPH receptor A5
EPHA6	NM_001080448	285220	EPH receptor A6
EPHA6	XM_114973	285220	EPH receptor A6
EPHA7	NM_004440	2045	EPH receptor A7
EPHA8	NM_001006943	2046	EPH receptor A8
EPHB1	NM_004441	2047	EPH receptor B1
EPHB2	NM_004442	2048	EPH receptor B2
EPHB3	NM_004443	2049	EPH receptor B3
EPHB4	NM_004444	2050	EPH receptor B4
EPHB6	NM_004445	2051	EPH receptor B6
ERBB2	NM_001005862	2064	v-erb-b2 erythroblastic leukemia viral oncogene homolog 2, neuro/glioblastom (avian)
ERBB3	NM_001005915	2065	v-erb-b2 erythroblastic leukemia viral oncogene homolog 3 (avian)
ERBB4	NM_001042599	2066	v-erb-a erythroblastic leukemia viral oncogene homolog 4 (avian)
ERBB4	NM_005235	2066	v-erb-a erythroblastic leukemia viral oncogene homolog 4 (avian)
ERN1	NM_001433	2081	endoplasmic reticulum to nucleus signaling 1
ERN1	NM_152461	2081	endoplasmic reticulum to nucleus signaling 1
ERN2	NM_033266	10595	endoplasmic reticulum to nucleus signaling 2
ETNK1	NM_001039481	55500	ethanolamine kinase 1
ETNK1	NM_018638	55500	ethanolamine kinase 1
ETNK2	NM_018208	55224	ethanolamine kinase 2
EVI5L	NM_145245	115704	ecotropic viral integration site 5-like
FASTK	NM_006712	10922	Fas-activated serine/threonine kinase
FER	NM_005246	2241	fer (fps/fes related) tyrosine kinase
FES	NM_002005	2242	feline sarcoma oncogene
FGFR1	NM_000604	2260	fibroblast growth factor receptor 1
FGFR1	NM_015850	2260	fibroblast growth factor receptor 1
FGFR2	NM_000141	2263	fibroblast growth factor receptor 2
FGFR2	NM_022974	2263	fibroblast growth factor receptor 2
FGFR2	NM_022976	2263	fibroblast growth factor receptor 2
FGFR3	NM_000142	2261	fibroblast growth factor receptor 3
FGFR4	NM_002011	2264	fibroblast growth factor receptor 4
FGGY	NM_001113411	55277	FGGY carbohydrate kinase domain containing
FGGY	NM_018291	55277	FGGY carbohydrate kinase domain containing
FGR	NM_001042729	2268	Gardner-Rasheed feline sarcoma viral (v-fgr) oncogene homolog
FGR	NM_005248	2268	Gardner-Rasheed feline sarcoma viral (v-fgr) oncogene homolog
FLJ25006	NM_144610	124923	uncharacterized serine/threonine-protein kinase SgK494
FLJ40852	NM_173677	285962	hypothetical LOC285962
FLT1	NM_002019	2321	fms-related tyrosine kinase 1 (vascular endothelial growth factor/vascular pern
FLT3	NM_004119	2322	fms-related tyrosine kinase 3
FLT4	NM_002020	2324	fms-related tyrosine kinase 4
FN3K	NM_022158	64122	fructosamine 3 kinase

FRAP1	NM_004958	2475	mechanistic target of rapamycin (serine/threonine kinase)
FRK	NM_002031	2444	fyn-related kinase
FUK	NM_145059	197258	fucokinase
FYN	NM_002037	2534	FYN oncogene related to SRC, FGR, YES
GAK	NM_005255	2580	cyclin G associated kinase
GALK1	NM_000154	2584	galactokinase 1
GALK2	NM_001001556	2585	galactokinase 2
GCK	NM_000162	2645	glucokinase (hexokinase 4)
GK	NM_000167	2710	glycerol kinase
GK2	NM_033214	2712	glycerol kinase 2
GNE	NM_001128227	10020	glucosamine (UDP-N-acetyl)-2-epimerase/N-acetylmannosamine kinase
GPR125	NM_145290	166647	G protein-coupled receptor 125
GRK1	NM_002929	6011	G protein-coupled receptor kinase 1
GRK4	NM_001004056	2868	G protein-coupled receptor kinase 4
GRK5	NM_005308	2869	G protein-coupled receptor kinase 5
GRK6	NM_001004105	2870	G protein-coupled receptor kinase 6
GRK7	NM_139209	131890	G protein-coupled receptor kinase 7
GSG2	NM_031965	83903	germ cell associated 2 (haspin)
GSK3A	NM_019884	2931	glycogen synthase kinase 3 alpha
GSK3B	NM_002093	2932	glycogen synthase kinase 3 beta
GUCY2C	NM_004963	2984	guanylate cyclase 2C (heat stable enterotoxin receptor)
GUCY2D	NM_000180	3000	guanylate cyclase 2D, membrane (retina-specific)
GUCY2F	NM_001522	2986	guanylate cyclase 2F, retinal
GUK1	NM_000858	2987	guanylate kinase 1
HCK	NM_002110	3055	hemopoietic cell kinase
HERC2	NM_004667	8924	hect domain and RLD 2
HIPK1	NM_152696	204851	homeodomain interacting protein kinase 1
HIPK2	NM_001113239	28996	homeodomain interacting protein kinase 2
HIPK2	NM_022740	28996	homeodomain interacting protein kinase 2
HIPK3	NM_001048200	10114	homeodomain interacting protein kinase 3
HIPK3	NM_005734	10114	homeodomain interacting protein kinase 3
HIPK4	NM_144685	147746	homeodomain interacting protein kinase 4
HK1	NM_000188	3098	hexokinase 1
HK2	NM_000189	3099	hexokinase 2
HK3	NM_002115	3101	hexokinase 3 (white cell)
HKDC1	NM_025130	80201	hexokinase domain containing 1
HSPB8	NM_014365	26353	heat shock 22kDa protein 8
HUNK	NM_014586	30811	hormonally up-regulated Neu-associated kinase
ICK	NM_014920	22858	intestinal cell (MAK-like) kinase
IGF1R	NM_000875	3480	insulin-like growth factor 1 receptor
IHPK1	NM_001006115	9807	inositol hexakisphosphate kinase 1
IHPK1	NM_153273	9807	inositol hexakisphosphate kinase 1
IHPK2	NM_001005909	51447	inositol hexakisphosphate kinase 2
IHPK2	NM_001005910	51447	inositol hexakisphosphate kinase 2
IHPK2	NM_001005912	51447	inositol hexakisphosphate kinase 2
IHPK3	NM_054111	117283	inositol hexakisphosphate kinase 3
IKBKB	NM_001556	3551	inhibitor of kappa light polypeptide gene enhancer in B-cells, kinase beta

IKBKE	NM_014002	9641	inhibitor of kappa light polypeptide gene enhancer in B-cells, kinase epsilon
IKBKG	NM_001099856	8517	inhibitor of kappa light polypeptide gene enhancer in B-cells, kinase gamma
IKBKG	NM_003639	8517	inhibitor of kappa light polypeptide gene enhancer in B-cells, kinase gamma
ILK	NM_001014794	3611	integrin-linked kinase
INSR	NM_000208	3643	insulin receptor
INSRR	NM_014215	3645	insulin receptor-related receptor
IPMK	NM_152230	253430	inositol polyphosphate multikinase
IRAK1	NM_001025242	3654	interleukin-1 receptor-associated kinase 1
IRAK2	NM_001570	3656	interleukin-1 receptor-associated kinase 2
IRAK3	NM_007199	11213	interleukin-1 receptor-associated kinase 3
IRAK4	NM_001114182	51135	interleukin-1 receptor-associated kinase 4
IRAK4	NM_016123	51135	interleukin-1 receptor-associated kinase 4
ITK	NM_005546	3702	IL2-inducible T-cell kinase
ITPK1	NM_014216	3705	inositol 1,3,4-triphosphate 5/6 kinase
ITPKA	NM_002220	3706	inositol 1,4,5-trisphosphate 3-kinase A
ITPKB	NM_002221	3707	inositol 1,4,5-trisphosphate 3-kinase B
ITPKC	NM_025194	80271	inositol 1,4,5-trisphosphate 3-kinase C
JAK1	NM_002227	3716	Janus kinase 1
JAK2	NM_004972	3717	Janus kinase 2
JAK3	NM_000215	3718	Janus kinase 3
KALRN	NM_001024660	8997	kalirin, RhoGEF kinase
KALRN	NM_003947	8997	kalirin, RhoGEF kinase
KDR	NM_002253	3791	kinase insert domain receptor (a type III receptor tyrosine kinase)
KHK	NM_000221	3795	ketohexokinase (fructokinase)
KIAA0999	NM_025164	23387	SIK family kinase 3
KIAA1804	NM_032435	84451	mixed lineage kinase 4
KIT	NM_000222	3815	v-kit Hardy-Zuckerman 4 feline sarcoma viral oncogene homolog
KSR1	NM_014238	8844	kinase suppressor of ras 1
KSR2	NM_173598	283455	kinase suppressor of ras 2
LATS1	NM_004690	9113	LATS, large tumor suppressor, homolog 1 (Drosophila)
LATS2	NM_014572	26524	LATS, large tumor suppressor, homolog 2 (Drosophila)
LCK	NM_005356	3932	lymphocyte-specific protein tyrosine kinase
LCK	NM_005356	3932	lymphocyte-specific protein tyrosine kinase
LCK	NM_001042771	3932	lymphocyte-specific protein tyrosine kinase
LIMK1	NM_002314	3984	LIM domain kinase 1
LIMK2	NM_001031801	3985	LIM domain kinase 2
LMTK2	NM_014916	22853	lemur tyrosine kinase 2
LMTK3	XM_055866	114783	lemur tyrosine kinase 3
LMTK3	XM_055866	114783	lemur tyrosine kinase 3
LMTK3	NM_001080434	114783	lemur tyrosine kinase 3
LOC390975	XM_372749	390975	
LOC442075	XM_001720051	442075	hypothetical LOC442075
LRGUK	NM_144648	136332	leucine-rich repeats and guanylate kinase domain containing
LRRK1	NM_024652	79705	leucine-rich repeat kinase 1
LRRK2	NM_198578	120892	leucine-rich repeat kinase 2
LTK	NM_002344	4058	leukocyte receptor tyrosine kinase
LYN	NM_002350	4067	v-yes-1 Yamaguchi sarcoma viral related oncogene homolog

LYN	NM_002350	4067	v-yes-1 Yamaguchi sarcoma viral related oncogene homolog
LYN	NM_001111097	4067	v-yes-1 Yamaguchi sarcoma viral related oncogene homolog
MAGI1	NM_001033057	9223	membrane associated guanylate kinase, WW and PDZ domain containing 1
MAGI2	NM_012301	9863	membrane associated guanylate kinase, WW and PDZ domain containing 2
MAK	NM_005906	4117	male germ cell-associated kinase
MAP2K1	NM_002755	5604	mitogen-activated protein kinase kinase 1
MAP2K2	NM_030662	5605	mitogen-activated protein kinase kinase 2
MAP2K3	NM_002756	5606	mitogen-activated protein kinase kinase 3
MAP2K4	NM_003010	6416	mitogen-activated protein kinase kinase 4
MAP2K5	NM_002757	5607	mitogen-activated protein kinase kinase 5
MAP2K6	NM_002758	5608	mitogen-activated protein kinase kinase 6
MAP2K7	NM_145185	5609	mitogen-activated protein kinase kinase 7
MAP3K1	NM_005921	4214	mitogen-activated protein kinase kinase kinase 1
MAP3K1	XM_042066	4214	mitogen-activated protein kinase kinase kinase 1
MAP3K10	NM_002446	4294	mitogen-activated protein kinase kinase kinase 10
MAP3K11	NM_002419	4296	mitogen-activated protein kinase kinase kinase 11
MAP3K12	NM_006301	7786	mitogen-activated protein kinase kinase kinase 12
MAP3K13	NM_004721	9175	mitogen-activated protein kinase kinase kinase 13
MAP3K14	NM_003954	9020	mitogen-activated protein kinase kinase kinase 14
MAP3K15	NM_001001671	389840	mitogen-activated protein kinase kinase kinase 15
MAP3K2	NM_006609	10746	mitogen-activated protein kinase kinase kinase 2
MAP3K3	NM_002401	4215	mitogen-activated protein kinase kinase kinase 3
MAP3K4	NM_005922	4216	mitogen-activated protein kinase kinase kinase 4
MAP3K5	NM_005923	4217	mitogen-activated protein kinase kinase kinase 5
MAP3K6	NM_004672	9064	mitogen-activated protein kinase kinase kinase 6
MAP3K7	NM_003188	6885	mitogen-activated protein kinase kinase kinase 7
MAP3K8	NM_005204	1326	mitogen-activated protein kinase kinase kinase 8
MAP3K9	NM_033141	4293	mitogen-activated protein kinase kinase kinase 9
MAP4K1	NM_001042600	11184	mitogen-activated protein kinase kinase kinase kinase 1
MAP4K1	NM_007181	11184	mitogen-activated protein kinase kinase kinase kinase 1
MAP4K2	NM_004579	5871	mitogen-activated protein kinase kinase kinase kinase 2
MAP4K3	NM_003618	8491	mitogen-activated protein kinase kinase kinase kinase 3
MAP4K4	NM_004834	9448	mitogen-activated protein kinase kinase kinase kinase 4
MAP4K5	NM_006575	11183	mitogen-activated protein kinase kinase kinase kinase 5
MAPK1	NM_002745	5594	mitogen-activated protein kinase 1
MAPK10	NM_002753	5602	mitogen-activated protein kinase 10
MAPK10	NM_138980	5602	mitogen-activated protein kinase 10
MAPK11	NM_002751	5600	mitogen-activated protein kinase 11
MAPK12	NM_002969	6300	mitogen-activated protein kinase 12
MAPK13	NM_002754	5603	mitogen-activated protein kinase 13
MAPK14	NM_001315	1432	mitogen-activated protein kinase 14
MAPK15	NM_139021	225689	mitogen-activated protein kinase 15
MAPK3	NM_001040056	5595	mitogen-activated protein kinase 3
MAPK4	NM_002747	5596	mitogen-activated protein kinase 4
MAPK6	NM_002748	5597	mitogen-activated protein kinase 6
MAPK7	NM_002749	5598	mitogen-activated protein kinase 7
MAPK8	NM_002750	5599	mitogen-activated protein kinase 8

MAPK9	NM_002752	5601	mitogen-activated protein kinase 9
MAPKAPK2	NM_004759	9261	mitogen-activated protein kinase-activated protein kinase 2
MAPKAPK2	NM_032960	9261	mitogen-activated protein kinase-activated protein kinase 2
MAPKAPK3	NM_004635	7867	mitogen-activated protein kinase-activated protein kinase 3
MAPKAPK5	NM_003668	8550	mitogen-activated protein kinase-activated protein kinase 5
MARK1	NM_018650	4139	MAP/microtubule affinity-regulating kinase 1
MARK2	NM_001039468	2011	MAP/microtubule affinity-regulating kinase 2
MARK3	NM_002376	4140	MAP/microtubule affinity-regulating kinase 3
MARK4	NM_031417	57787	MAP/microtubule affinity-regulating kinase 4
MARVELD3	NM_001017967	91862	MARVEL domain containing 3
MARVELD3	NM_052858	91862	MARVEL domain containing 3
MAST1	NM_014975	22983	microtubule associated serine/threonine kinase 1
MAST2	NM_015112	23139	microtubule associated serine/threonine kinase 2
MAST3	NM_015016	23031	microtubule associated serine/threonine kinase 3
MAST3	NM_015016	23031	microtubule associated serine/threonine kinase 3
MAST3	NM_015016	23031	microtubule associated serine/threonine kinase 3
MAST4	NM_015183	375449	microtubule associated serine/threonine kinase family member 4
MAST4	NM_198828	375449	microtubule associated serine/threonine kinase family member 4
MASTL	NM_032844	84930	microtubule associated serine/threonine kinase-like
MATK	NM_002378	4145	megakaryocyte-associated tyrosine kinase
MELK	NM_014791	9833	maternal embryonic leucine zipper kinase
MERTK	NM_006343	10461	c-mer proto-oncogene tyrosine kinase
MET	NM_000245	4233	met proto-oncogene (hepatocyte growth factor receptor)
MGC16169	NM_033115	93627	TBC1 domain containing kinase
MGC42105	NM_153361	167359	serine/threonine-protein kinase NIM1
MINK1	NM_001024937	50488	misshapen-like kinase 1 (zebrafish)
MIP	NM_012064	4284	major intrinsic protein of lens fiber
MKNK1	NM_003684	8569	MAP kinase interacting serine/threonine kinase 1
MKNK1	NM_003684	8569	MAP kinase interacting serine/threonine kinase 1
MKNK1	NM_003684	8569	MAP kinase interacting serine/threonine kinase 1
MKNK2	NM_017572	2872	MAP kinase interacting serine/threonine kinase 2
MLKL	NM_152649	197259	mixed lineage kinase domain-like
MOS	NM_005372	4342	v-mos Moloney murine sarcoma viral oncogene homolog
MPP1	NM_002436	4354	membrane protein, palmitoylated 1, 55kDa
MPP2	NM_005374	4355	membrane protein, palmitoylated 2 (MAGUK p55 subfamily member 2)
MPP3	NM_001932	4356	membrane protein, palmitoylated 3 (MAGUK p55 subfamily member 3)
MPP4	NM_033066	58538	membrane protein, palmitoylated 4 (MAGUK p55 subfamily member 4)
MPP5	NM_022474	64398	membrane protein, palmitoylated 5 (MAGUK p55 subfamily member 5)
MPP6	NM_016447	51678	membrane protein, palmitoylated 6 (MAGUK p55 subfamily member 6)
MST1R	NM_002447	4486	macrophage stimulating 1 receptor (c-met-related tyrosine kinase)
MUSK	NM_005592	4593	muscle, skeletal, receptor tyrosine kinase
MVK	NM_000431	4598	mevalonate kinase
MYLK	NM_053025	4638	myosin light chain kinase
MYLK	NM_053025	4638	myosin light chain kinase
MYLK	NM_005965	4638	myosin light chain kinase
MYLK	NM_053029	4638	myosin light chain kinase
MYLK	NM_053031	4638	myosin light chain kinase

MYLK	NM_053032	4638	myosin light chain kinase
MYLK2	NM_033118	85366	myosin light chain kinase 2
MYLK3	NM_182493	91807	myosin light chain kinase 3
MYLK4	NM_001012418	340156	myosin light chain kinase family, member 4
MYO3A	NM_017433	53904	myosin IIIA
MYO3B	NM_001083615	140469	myosin IIIB
MYO3B	NM_138995	140469	myosin IIIB
NAALADL1	NM_005468	10004	N-acetylated alpha-linked acidic dipeptidase-like 1
NADK	NM_023018	65220	NAD kinase
NAGK	NM_017567	55577	N-acetylglucosamine kinase
NDUFA10	NM_004544	4705	NADH dehydrogenase (ubiquinone) 1 alpha subcomplex, 10, 42kDa
NEK1	NM_012224	4750	NIMA (never in mitosis gene a)-related kinase 1
NEK10	NM_001031741	152110	NIMA (never in mitosis gene a)- related kinase 10
NEK11	NM_024800	79858	NIMA (never in mitosis gene a)- related kinase 11
NEK2	NM_002497	4751	NIMA (never in mitosis gene a)-related kinase 2
NEK3	NM_002498	4752	NIMA (never in mitosis gene a)-related kinase 3
NEK4	NM_003157	6787	NIMA (never in mitosis gene a)-related kinase 4
NEK5	NM_199289	341676	NIMA (never in mitosis gene a)-related kinase 5
NEK6	NM_014397	10783	NIMA (never in mitosis gene a)-related kinase 6
NEK7	NM_133494	140609	NIMA (never in mitosis gene a)-related kinase 7
NEK8	NM_178170	284086	NIMA (never in mitosis gene a)- related kinase 8
NEK9	NM_033116	91754	NIMA (never in mitosis gene a)- related kinase 9
NLK	NM_016231	51701	nemo-like kinase
NME1	NM_000269	4830	non-metastatic cells 1, protein (NM23A) expressed in
NME1-NME2	NM_001018136	654364	NME1-NME2 readthrough transcript
NME3	NM_002513	4832	non-metastatic cells 3, protein expressed in
NME4	NM_005009	4833	non-metastatic cells 4, protein expressed in
NME5	NM_003551	8382	non-metastatic cells 5, protein expressed in (nucleoside-diphosphate kinase)
NME6	NM_005793	10201	non-metastatic cells 6, protein expressed in (nucleoside-diphosphate kinase)
NME7	NM_013330	29922	non-metastatic cells 7, protein expressed in (nucleoside-diphosphate kinase)
NPR1	NM_000906	4881	natriuretic peptide receptor A/guanylate cyclase A (atrionatriuretic peptide rece
NPR2	NM_003995	4882	natriuretic peptide receptor B/guanylate cyclase B (atrionatriuretic peptide rece
NRBP1	NM_013392	29959	nuclear receptor binding protein 1
NRBP2	NM_178564	340371	nuclear receptor binding protein 2
NRK	NM_198465	203447	Nik related kinase
NTRK1	NM_001007792	4914	neurotrophic tyrosine kinase, receptor, type 1
NTRK2	NM_001007097	4915	neurotrophic tyrosine kinase, receptor, type 2
NTRK3	NM_001007156	4916	neurotrophic tyrosine kinase, receptor, type 3
NUAK1	NM_014840	9891	NUAK family, SNF1-like kinase, 1
NUAK2	NM_030952	81788	NUAK family, SNF1-like kinase, 2
OBSCN	NM_001098623	84033	obscurin, cytoskeletal calmodulin and titin-interacting RhoGEF
OBSCN	NM_052843	84033	obscurin, cytoskeletal calmodulin and titin-interacting RhoGEF
OXSM	NM_017897	54995	3-oxoacyl-ACP synthase, mitochondrial
OXSR1	NM_005109	9943	oxidative-stress responsive 1
PAK1	NM_002576	5058	p21 protein (Cdc42/Rac)-activated kinase 1
PAK2	NM_002577	5062	p21 protein (Cdc42/Rac)-activated kinase 2
PAK3	NM_001128167	5063	p21 protein (Cdc42/Rac)-activated kinase 3

PAK3	NM_001128166	5063	p21 protein (Cdc42/Rac)-activated kinase 3
PAK4	NM_001014834	10298	p21 protein (Cdc42/Rac)-activated kinase 4
PAK4	NM_001014831	10298	p21 protein (Cdc42/Rac)-activated kinase 4
PAK6	NM_020168	56924	p21 protein (Cdc42/Rac)-activated kinase 6
PAK7	NM_177990	57144	p21 protein (Cdc42/Rac)-activated kinase 7
PAK7	NM_020341	57144	p21 protein (Cdc42/Rac)-activated kinase 7
PAN3	NM_175854	255967	PAN3 poly(A) specific ribonuclease subunit homolog (S. cerevisiae)
PANK1	NM_138316	53354	pantothenate kinase 1
PANK1	NM_148977	53354	pantothenate kinase 1
PANK3	NM_024594	79646	pantothenate kinase 3
PAPSS1	NM_005443	9061	3'-phosphoadenosine 5'-phosphosulfate synthase 1
PAPSS2	NM_001015880	9060	3'-phosphoadenosine 5'-phosphosulfate synthase 2
PASK	NM_015148	23178	PAS domain containing serine/threonine kinase
PBK	NM_018492	55872	PDZ binding kinase
PCK1	NM_002591	5105	phosphoenolpyruvate carboxykinase 1 (soluble)
PCK2	NM_001018073	5106	phosphoenolpyruvate carboxykinase 2 (mitochondrial)
PCTK1	NM_006201	5127	PCTAIRE protein kinase 1
PCTK2	NM_002595	5128	PCTAIRE protein kinase 2
PCTK3	NM_002596	5129	PCTAIRE protein kinase 3
PDGFRA	NM_006206	5156	platelet-derived growth factor receptor, alpha polypeptide
PDGFRB	NM_002609	5159	platelet-derived growth factor receptor, beta polypeptide
PDIK1L	NM_152835	149420	PDLIM1 interacting kinase 1 like
PDK1	NM_002610	5163	pyruvate dehydrogenase kinase, isozyme 1
PDK2	NM_002611	5164	pyruvate dehydrogenase kinase, isozyme 2
PDK3	NM_005391	5165	pyruvate dehydrogenase kinase, isozyme 3
PDK4	NM_002612	5166	pyruvate dehydrogenase kinase, isozyme 4
PDPK1	NM_002613	5170	3-phosphoinositide dependent protein kinase-1
PDXK	NM_003681	8566	pyridoxal (pyridoxine, vitamin B6) kinase
PDZD2	NM_178140	23037	PDZ domain containing 2
PFKFB1	NM_002625	5207	6-phosphofructo-2-kinase/fructose-2,6-biphosphatase 1
PFKFB2	NM_001018053	5208	6-phosphofructo-2-kinase/fructose-2,6-biphosphatase 2
PFKFB3	NM_004566	5209	6-phosphofructo-2-kinase/fructose-2,6-biphosphatase 3
PFKFB4	NM_004567	5210	6-phosphofructo-2-kinase/fructose-2,6-biphosphatase 4
PFKFB4	NM_004567	5210	6-phosphofructo-2-kinase/fructose-2,6-biphosphatase 4
PFKL	NM_001002021	5211	phosphofructokinase, liver
PFKM	NM_000289	5213	phosphofructokinase, muscle
PFKP	NM_002627	5214	phosphofructokinase, platelet
PFTK1	NM_012395	5218	PFTAIRE protein kinase 1
PFTK2	NM_139158	65061	PFTAIRE protein kinase 2
PGK1	NM_000291	5230	phosphoglycerate kinase 1
PGK2	NM_138733	5232	phosphoglycerate kinase 2
PHKA1	NM_001122670	5255	phosphorylase kinase, alpha 1 (muscle)
PHKA1	NM_002637	5255	phosphorylase kinase, alpha 1 (muscle)
PHKA2	NM_000292	5256	phosphorylase kinase, alpha 2 (liver)
PHKB	NM_000293	5257	phosphorylase kinase, beta
PHKG1	NM_006213	5260	phosphorylase kinase, gamma 1 (muscle)
PHKG2	NM_000294	5261	phosphorylase kinase, gamma 2 (testis)

PI4K2A	NM_018425	55361	phosphatidylinositol 4-kinase type 2 alpha
PI4K2B	NM_018323	55300	phosphatidylinositol 4-kinase type 2 beta
PI4KA	NM_002650	5297	phosphatidylinositol 4-kinase, catalytic, alpha
PI4KA	XM_001721947	5297	phosphatidylinositol 4-kinase, catalytic, alpha
PI4KB	NM_002651	5298	phosphatidylinositol 4-kinase, catalytic, beta
PIK3C2A	NM_002645	5286	phosphoinositide-3-kinase, class 2, alpha polypeptide
PIK3C2B	NM_002646	5287	phosphoinositide-3-kinase, class 2, beta polypeptide
PIK3C2G	NM_004570	5288	phosphoinositide-3-kinase, class 2, gamma polypeptide
PIK3C3	NM_002647	5289	phosphoinositide-3-kinase, class 3
PIK3CA	NM_006218	5290	phosphoinositide-3-kinase, catalytic, alpha polypeptide
PIK3CB	NM_006219	5291	phosphoinositide-3-kinase, catalytic, beta polypeptide
PIK3CD	NM_005026	5293	phosphoinositide-3-kinase, catalytic, delta polypeptide
PIK3CG	NM_002649	5294	phosphoinositide-3-kinase, catalytic, gamma polypeptide
PIK3R2	NM_005027	5296	phosphoinositide-3-kinase, regulatory subunit 2 (beta)
PIK3R3	NM_001114172	8503	phosphoinositide-3-kinase, regulatory subunit 3 (gamma)
PIK3R3	NM_003629	8503	phosphoinositide-3-kinase, regulatory subunit 3 (gamma)
PIK3R4	NM_014602	30849	phosphoinositide-3-kinase, regulatory subunit 4
PIM1	NM_002648	5292	pim-1 oncogene
PIM2	NM_006875	11040	pim-2 oncogene
PIM3	NM_001001852	415116	pim-3 oncogene
PIN1	NM_006221	5300	peptidylprolyl cis/trans isomerase, NIMA-interacting 1
PINK1	NM_032409	65018	PTEN induced putative kinase 1
PIP4K2A	NM_005028	5305	phosphatidylinositol-5-phosphate 4-kinase, type II, alpha
PIP4K2B	NM_003559	8396	phosphatidylinositol-5-phosphate 4-kinase, type II, beta
PIP4K2C	NM_024779	79837	phosphatidylinositol-5-phosphate 4-kinase, type II, gamma
PIP5K1A	NM_003557	8394	phosphatidylinositol-4-phosphate 5-kinase, type I, alpha
PIP5K1B	NM_001031687	8395	phosphatidylinositol-4-phosphate 5-kinase, type I, beta
PIP5K1B	NM_003558	8395	phosphatidylinositol-4-phosphate 5-kinase, type I, beta
PIP5K1C	NM_012398	23396	phosphatidylinositol-4-phosphate 5-kinase, type I, gamma
PIP5K3	NM_001002881	200576	phosphoinositide kinase, FYVE finger containing
PIP5K3	NM_015040	200576	phosphoinositide kinase, FYVE finger containing
PKD2L1	NM_016112	9033	polycystic kidney disease 2-like 1
PKD2L2	NM_014386	27039	polycystic kidney disease 2-like 2
PKLR	NM_000298	5313	pyruvate kinase, liver and RBC
PKM2	NM_002654	5315	pyruvate kinase, muscle
PKMYT1	NM_004203	9088	protein kinase, membrane associated tyrosine/threonine 1
PKN1	NM_002741	5585	protein kinase N1
PKN2	NM_006256	5586	protein kinase N2
PKN3	NM_013355	29941	protein kinase N3
PLK1	NM_005030	5347	polo-like kinase 1 (Drosophila)
PLK2	NM_006622	10769	polo-like kinase 2 (Drosophila)
PLK3	NM_004073	1263	polo-like kinase 3 (Drosophila)
PLK4	NM_014264	10733	polo-like kinase 4 (Drosophila)
PLK5P	XR_015543	126520	polo-like kinase 5 pseudogene
PLK5P	XM_927549	126520	polo-like kinase 5 pseudogene
PLK5P	XR_015464	126520	polo-like kinase 5 pseudogene
PMVK	NM_006556	10654	phosphomevalonate kinase

PNCK	NM_001039582	139728	pregnancy up-regulated non-ubiquitously expressed CaM kinase
POLK	NM_016218	51426	polymerase (DNA directed) kappa
POLR2K	NM_005034	5440	polymerase (RNA) II (DNA directed) polypeptide K, 7.0kDa
POLR3K	NM_016310	51728	polymerase (RNA) III (DNA directed) polypeptide K, 12.3 kDa
PRKAA1	NM_006251	5562	protein kinase, AMP-activated, alpha 1 catalytic subunit
PRKAA2	NM_006252	5563	protein kinase, AMP-activated, alpha 2 catalytic subunit
PRKAB1	NM_006253	5564	protein kinase, AMP-activated, beta 1 non-catalytic subunit
PRKAB2	NM_005399	5565	protein kinase, AMP-activated, beta 2 non-catalytic subunit
PRKACA	NM_002730	5566	protein kinase, cAMP-dependent, catalytic, alpha
PRKACB	NM_002731	5567	protein kinase, cAMP-dependent, catalytic, beta
PRKACG	NM_002732	5568	protein kinase, cAMP-dependent, catalytic, gamma
PRKAG1	NM_002733	5571	protein kinase, AMP-activated, gamma 1 non-catalytic subunit
PRKAG2	NM_001040633	51422	protein kinase, AMP-activated, gamma 2 non-catalytic subunit
PRKAG3	NM_017431	53632	protein kinase, AMP-activated, gamma 3 non-catalytic subunit
PRKAR1A	NM_002734	5573	protein kinase, cAMP-dependent, regulatory, type I, alpha
PRKAR1B	NM_002735	5575	protein kinase, cAMP-dependent, regulatory, type I, beta
PRKAR2A	NM_004157	5576	protein kinase, cAMP-dependent, regulatory, type II, alpha
PRKAR2B	NM_002736	5577	protein kinase, cAMP-dependent, regulatory, type II, beta
PRKCA	NM_002737	5578	protein kinase C, alpha
PRKCB	NM_002738	5579	protein kinase C, beta
PRKCD	NM_006254	5580	protein kinase C, delta
PRKCE	NM_005400	5581	protein kinase C, epsilon
PRKCG	NM_002739	5582	protein kinase C, gamma
PRKCH	NM_006255	5583	protein kinase C, eta
PRKCI	NM_002740	5584	protein kinase C, iota
PRKCQ	NM_006257	5588	protein kinase C, theta
PRKCZ	NM_001033581	5590	protein kinase C, zeta
PRKD1	NM_002742	5587	protein kinase D1
PRKD2	NM_001079880	25865	protein kinase D2
PRKD2	NM_016457	25865	protein kinase D2
PRKD3	NM_005813	23683	protein kinase D3
PRKDC	NM_006904	5591	protein kinase, DNA-activated, catalytic polypeptide
PRKDC	NM_001081640	5591	protein kinase, DNA-activated, catalytic polypeptide
PRKG1	NM_006258	5592	protein kinase, cGMP-dependent, type I
PRKG1	NM_001098512	5592	protein kinase, cGMP-dependent, type I
PRKG2	NM_006259	5593	protein kinase, cGMP-dependent, type II
PRKX	NM_005044	5613	protein kinase, X-linked
PRKY	NM_002760	5616	protein kinase, Y-linked
PRPF4B	NM_003913	8899	PRP4 pre-mRNA processing factor 4 homolog B (yeast)
PRPS1	NM_002764	5631	phosphoribosyl pyrophosphate synthetase 1
PRPS1L1	NM_175886	221823	phosphoribosyl pyrophosphate synthetase 1-like 1
PRPS2	NM_001039091	5634	phosphoribosyl pyrophosphate synthetase 2
PSKH1	NM_006742	5681	protein serine kinase H1
PSKH2	NM_033126	85481	protein serine kinase H2
PTK2	NM_005607	5747	PTK2 protein tyrosine kinase 2
PTK2B	NM_004103	2185	PTK2B protein tyrosine kinase 2 beta
PTK6	NM_005975	5753	PTK6 protein tyrosine kinase 6

PTK7	NM_002821	5754	PTK7 protein tyrosine kinase 7
PTK7	NM_152883	5754	PTK7 protein tyrosine kinase 7
PXK	NM_017771	54899	PX domain containing serine/threonine kinase
RAB32	NM_006834	10981	RAB32, member RAS oncogene family
RAB38	NM_022337	23682	RAB38, member RAS oncogene family
RAD18	NM_020165	56852	RAD18 homolog (S. cerevisiae)
RAF1	NM_002880	5894	v-raf-1 murine leukemia viral oncogene homolog 1
RAGE	NM_014226	5891	renal tumor antigen
RALB	NM_002881	5899	v-ral simian leukemia viral oncogene homolog B
RAPGEF3	NM_006105	10411	Rap guanine nucleotide exchange factor (GEF) 3
RAPGEF3	NM_001098531	10411	Rap guanine nucleotide exchange factor (GEF) 3
RAPGEF4	NM_001100397	11069	Rap guanine nucleotide exchange factor (GEF) 4
RAPGEF4	NM_007023	11069	Rap guanine nucleotide exchange factor (GEF) 4
RBKS	NM_022128	64080	ribokinase
RET	NM_020630	5979	ret proto-oncogene
RFK	NM_018339	55312	riboflavin kinase
RIOK1	NM_031480	83732	RIO kinase 1 (yeast)
RIOK2	NM_018343	55781	RIO kinase 2 (yeast)
RIOK3	NM_003831	8780	RIO kinase 3 (yeast)
RIOK3	NM_145906	8780	RIO kinase 3 (yeast)
RIPK1	NM_003804	8737	receptor (TNFRSF)-interacting serine-threonine kinase 1
RIPK2	NM_003821	8767	receptor-interacting serine-threonine kinase 2
RIPK3	NM_006871	11035	receptor-interacting serine-threonine kinase 3
RIPK4	NM_020639	54101	receptor-interacting serine-threonine kinase 4
RIPK5	NM_015375	25778	dual serine/threonine and tyrosine protein kinase
RNASEL	NM_021133	6041	ribonuclease L (2',5'-oligoisoadenylate synthetase-dependent)
ROCK1	NM_005406	6093	Rho-associated, coiled-coil containing protein kinase 1
ROCK2	NM_004850	9475	Rho-associated, coiled-coil containing protein kinase 2
ROR1	NM_001083592	4919	receptor tyrosine kinase-like orphan receptor 1
ROR1	NM_005012	4919	receptor tyrosine kinase-like orphan receptor 1
ROR2	NM_004560	4920	receptor tyrosine kinase-like orphan receptor 2
ROS1	NM_002944	6098	c-ros oncogene 1 , receptor tyrosine kinase
RP6-213H19.1	NM_001042452	51765	serine/threonine protein kinase MST4
RP6-213H19.1	NM_016542	51765	serine/threonine protein kinase MST4
RPS6KA1	NM_001006665	6195	ribosomal protein S6 kinase, 90kDa, polypeptide 1
RPS6KA2	NM_001006932	6196	ribosomal protein S6 kinase, 90kDa, polypeptide 2
RPS6KA3	NM_004586	6197	ribosomal protein S6 kinase, 90kDa, polypeptide 3
RPS6KA4	NM_001006944	8986	ribosomal protein S6 kinase, 90kDa, polypeptide 4
RPS6KA5	NM_004755	9252	ribosomal protein S6 kinase, 90kDa, polypeptide 5
RPS6KA6	NM_014496	27330	ribosomal protein S6 kinase, 90kDa, polypeptide 6
RPS6KB1	NM_003161	6198	ribosomal protein S6 kinase, 70kDa, polypeptide 1
RPS6KB2	NM_001007071	6199	ribosomal protein S6 kinase, 70kDa, polypeptide 2
RPS6KB2	NM_003952	6199	ribosomal protein S6 kinase, 70kDa, polypeptide 2
RPS6KC1	NM_012424	26750	ribosomal protein S6 kinase, 52kDa, polypeptide 1
RPS6KL1	NM_031464	83694	ribosomal protein S6 kinase-like 1
RYK	NM_001005861	6259	RYK receptor-like tyrosine kinase

SBK1	NM_001024401	388228	SH3-binding domain kinase 1
SCYL1	NM_001048218	57410	SCY1-like 1 (S. cerevisiae)
SCYL1	NM_020680	57410	SCY1-like 1 (S. cerevisiae)
SCYL2	NM_017988	55681	SCY1-like 2 (S. cerevisiae)
SCYL3	NM_020423	57147	SCY1-like 3 (S. cerevisiae)
SGK1	NM_005627	6446	serum/glucocorticoid regulated kinase 1
SGK196	NM_032237	84197	protein kinase-like protein SgK196
SGK2	NM_016276	10110	serum/glucocorticoid regulated kinase 2
SGK269	NM_024776	79834	NKF3 kinase family member
SGK269	XM_370878	79834	NKF3 kinase family member
SGK269	XM_935078	79834	NKF3 kinase family member
SGK3	NM_001033578	23678	serum/glucocorticoid regulated kinase family, member 3
SGK493	NM_138370	91461	protein kinase-like protein SgK493
SHPK	NM_013276	23729	sedoheptulokinase
SKP1	NM_006930	6500	S-phase kinase-associated protein 1
SKP1	NM_170679	6500	S-phase kinase-associated protein 1
SLK	NM_014720	9748	STE20-like kinase (yeast)
SMG1	NM_015092	23049	SMG1 homolog, phosphatidylinositol 3-kinase-related kinase (C. elegans)
SNF1LK	NM_173354	150094	salt-inducible kinase 1
SNF1LK2	NM_015191	23235	salt-inducible kinase 2
SNRK	NM_017719	54861	SNF related kinase
SNRK	NM_001100594	54861	SNF related kinase
SPEG	NM_005876	10290	SPEG complex locus
SPHK1	NM_021972	8877	sphingosine kinase 1
SPHK2	NM_020126	56848	sphingosine kinase 2
SRC	NM_005417	6714	v-src sarcoma (Schmidt-Ruppin A-2) viral oncogene homolog (avian)
SRMS	NM_080823	6725	src-related kinase lacking C-terminal regulatory tyrosine and N-terminal myrist
SRPK1	NM_003137	6732	SFRS protein kinase 1
SRPK2	NM_182691	6733	SFRS protein kinase 2
SRPK3	NM_014370	26576	SFRS protein kinase 3
STC1	NM_003155	6781	stanniocalcin 1
STK10	NM_005990	6793	serine/threonine kinase 10
STK11	NM_000455	6794	serine/threonine kinase 11
STK16	NM_001008910	8576	serine/threonine kinase 16
STK16	NM_003691	8576	serine/threonine kinase 16
STK17A	NM_004760	9263	serine/threonine kinase 17a
STK17B	NM_004226	9262	serine/threonine kinase 17b
STK19	NM_004197	8859	serine/threonine kinase 19
STK24	NM_001032296	8428	serine/threonine kinase 24 (STE20 homolog, yeast)
STK25	NM_006374	10494	serine/threonine kinase 25 (STE20 homolog, yeast)
STK3	NM_006281	6788	serine/threonine kinase 3 (STE20 homolog, yeast)
STK31	NM_001122833	56164	serine/threonine kinase 31
STK31	NM_031414	56164	serine/threonine kinase 31
STK32A	NM_001112724	202374	serine/threonine kinase 32A
STK32A	NM_145001	202374	serine/threonine kinase 32A
STK32B	NM_018401	55351	serine/threonine kinase 32B
STK32C	NM_173575	282974	serine/threonine kinase 32C

STK33	NM_030906	65975	serine/threonine kinase 33
STK35	NM_080836	140901	serine/threonine kinase 35
STK36	NM_015690	27148	serine/threonine kinase 36, fused homolog (Drosophila)
STK38	NM_007271	11329	serine/threonine kinase 38
STK38L	NM_015000	23012	serine/threonine kinase 38 like
STK39	NM_013233	27347	serine threonine kinase 39 (STE20/SPS1 homolog, yeast)
STK4	NM_006282	6789	serine/threonine kinase 4
STK40	NM_032017	83931	serine/threonine kinase 40
STRADA	NM_001003786	92335	STE20-related kinase adaptor alpha
STRADA	NM_153335	92335	STE20-related kinase adaptor alpha
STRADB	NM_018571	55437	STE20-related kinase adaptor beta
STYK1	NM_018423	55359	serine/threonine/tyrosine kinase 1
SYK	NM_003177	6850	spleen tyrosine kinase
TAF1	NM_004606	6872	TAF1 RNA polymerase II, TATA box binding protein (TBP)-associated factor, [illegible]
TAF1L	NM_153809	138474	TAF1 RNA polymerase II, TATA box binding protein (TBP)-associated factor, [illegible]
TAF9	NM_001015891	6880	TAF9 RNA polymerase II, TATA box binding protein (TBP)-associated factor, [illegible]
TAF9	NM_001015892	6880	TAF9 RNA polymerase II, TATA box binding protein (TBP)-associated factor, [illegible]
TANK	NM_004180	10010	TRAF family member-associated NFKB activator
TANK	NM_133484	10010	TRAF family member-associated NFKB activator
TAOK1	NM_020791	57551	TAO kinase 1
TAOK2	NM_004783	9344	TAO kinase 2
TAOK3	NM_016281	51347	TAO kinase 3
TBK1	NM_013254	29110	TANK-binding kinase 1
TCEB3C	NM_145653	162699	transcription elongation factor B polypeptide 3C (elongin A3)
TEC	NM_003215	7006	tec protein tyrosine kinase
TEK	NM_000459	7010	TEK tyrosine kinase, endothelial
TESK1	NM_006285	7016	testis-specific kinase 1
TESK2	NM_007170	10420	testis-specific kinase 2
TEX14	NM_031272	56155	testis expressed 14
TGFBR1	NM_004612	7046	transforming growth factor, beta receptor 1
TGFBR2	NM_001024847	7048	transforming growth factor, beta receptor II (70/80kDa)
TIE1	NM_005424	7075	tyrosine kinase with immunoglobulin-like and EGF-like domains 1
TJP1	NM_003257	7082	tight junction protein 1 (zona occludens 1)
TJP2	NM_004817	9414	tight junction protein 2 (zona occludens 2)
TJP3	NM_014428	27134	tight junction protein 3 (zona occludens 3)
TK1	NM_003258	7083	thymidine kinase 1, soluble
TK2	NM_004614	7084	thymidine kinase 2, mitochondrial
TLK1	NM_012290	9874	tousled-like kinase 1
TLK2	NM_001112707	11011	tousled-like kinase 2
TLK2	NM_006852	11011	tousled-like kinase 2
TNIK	NM_015028	23043	TRAF2 and NCK interacting kinase
TNK1	NM_003985	8711	tyrosine kinase, non-receptor, 1
TNK2	NM_001010938	10188	tyrosine kinase, non-receptor, 2
TNNI3K	NM_001112808	51086	TNNI3 interacting kinase
TNNI3K	NM_015978	51086	TNNI3 interacting kinase
TP53RK	NM_033550	112858	TP53 regulating kinase
TPK1	NM_001042482	27010	thiamin pyrophosphokinase 1

TPK1	NM_022445	27010	thiamin pyrophosphokinase 1
TRIB1	NM_025195	10221	tribbles homolog 1 (Drosophila)
TRIB2	NM_021643	28951	tribbles homolog 2 (Drosophila)
TRIB3	NM_021158	57761	tribbles homolog 3 (Drosophila)
TRIM24	NM_003852	8805	tripartite motif-containing 24
TRIM24	NM_015905	8805	tripartite motif-containing 24
TRIM28	NM_005762	10155	tripartite motif-containing 28
TRIM33	NM_015906	51592	tripartite motif-containing 33
TRIO	NM_007118	7204	triple functional domain (PTPRF interacting)
TRPM6	NM_017662	140803	transient receptor potential cation channel, subfamily M, member 6
TRPM7	NM_017672	54822	transient receptor potential cation channel, subfamily M, member 7
TRRAP	NM_003496	8295	transformation/transcription domain-associated protein
TSSK1B	NM_032028	83942	testis-specific serine kinase 1B
TSSK2	NM_053006	23617	testis-specific serine kinase 2
TSSK3	NM_052841	81629	testis-specific serine kinase 3
TSSK4	NM_174944	283629	testis-specific serine kinase 4
TSSK6	NM_032037	83983	testis-specific serine kinase 6
TTBK1	NM_032538	84630	tau tubulin kinase 1
TTBK2	NM_173500	146057	tau tubulin kinase 2
TTK	NM_003318	7272	TTK protein kinase
TTN	NM_133378	7273	titin
TTN	NM_003319	7273	titin
TWF1	NM_002822	5756	twinfilin, actin-binding protein, homolog 1 (Drosophila)
TWF2	NM_007284	11344	twinfilin, actin-binding protein, homolog 2 (Drosophila)
TXK	NM_003328	7294	TXK tyrosine kinase
TXNDC3	NM_016616	51314	thioredoxin domain containing 3 (spermatozoa)
TXNDC6	NM_178130	347736	thioredoxin domain containing 6
TYK2	NM_003331	7297	tyrosine kinase 2
TYRO3	NM_006293	7301	TYRO3 protein tyrosine kinase
UCK1	NM_031432	83549	uridine-cytidine kinase 1
UCK2	NM_012474	7371	uridine-cytidine kinase 2
UCKL1	NM_017859	54963	uridine-cytidine kinase 1-like 1
UHMK1	NM_175866	127933	U2AF homology motif (UHM) kinase 1
ULK1	NM_003565	8408	unc-51-like kinase 1 (C. elegans)
ULK2	NM_014683	9706	unc-51-like kinase 2 (C. elegans)
ULK3	NM_001099436	25989	unc-51-like kinase 3 (C. elegans)
ULK4	XM_929989	54986	unc-51-like kinase 4 (C. elegans)
ULK4	XM_929989	54986	unc-51-like kinase 4 (C. elegans)
ULK4	XM_934169	54986	unc-51-like kinase 4 (C. elegans)
ULK4	XM_934169	54986	unc-51-like kinase 4 (C. elegans)
ULK4	NM_017886	54986	unc-51-like kinase 4 (C. elegans)
ULK4	XM_934158	54986	unc-51-like kinase 4 (C. elegans)
ULK4	XM_934164	54986	unc-51-like kinase 4 (C. elegans)
ULK4	XM_934168	54986	unc-51-like kinase 4 (C. elegans)
ULK4	XM_934170	54986	unc-51-like kinase 4 (C. elegans)
ULK4	XM_934171	54986	unc-51-like kinase 4 (C. elegans)
VRK1	NM_003384	7443	vaccinia related kinase 1

VRK2	NM_006296	7444	vaccinia related kinase 2
VRK3	NM_001025778	51231	vaccinia related kinase 3
WEE1	NM_003390	7465	WEE1 homolog (S. pombe)
WEE2	NM_001105558	494551	WEE1 homolog 2 (S. pombe)
WNK1	NM_018979	65125	WNK lysine deficient protein kinase 1
WNK2	NM_006648	65268	WNK lysine deficient protein kinase 2
WNK3	NM_001002838	65267	WNK lysine deficient protein kinase 3
WNK4	NM_032387	65266	WNK lysine deficient protein kinase 4
XYLB	NM_005108	9942	xylulokinase homolog (H. influenzae)
YES1	NM_005433	7525	v-yes-1 Yamaguchi sarcoma viral oncogene homolog 1
YSK4	NM_001018046	80122	YSK4 Sps1/Ste20-related kinase homolog (S. cerevisiae)
ZAK	NM_016653	51776	sterile alpha motif and leucine zipper containing kinase AZK
ZAP70	NM_001079	7535	zeta-chain (TCR) associated protein kinase 70kDa
